# Epidemic Spreading Model to Characterize Misfolded Proteins Propagation in Aging and Associated Neurodegenerative Disorders

**DOI:** 10.1371/journal.pcbi.1003956

**Published:** 2014-11-20

**Authors:** Yasser Iturria-Medina, Roberto C. Sotero, Paule J. Toussaint, Alan C. Evans, and the Alzheimer's Disease Neuroimaging Initiative

**Affiliations:** Montreal Neurological Institute, Montreal, Quebec, Canada; Indiana University, United States of America

## Abstract

Misfolded proteins (MP) are a key component in aging and associated neurodegenerative disorders. For example, misfolded Amyloid-ß (Aß) and tau proteins are two neuropathogenic hallmarks of Alzheimer's disease. Mechanisms underlying intra-brain MP propagation/deposition remain essentially uncharacterized. Here, is introduced an epidemic spreading model (ESM) for MP dynamics that considers propagation-like interactions between MP agents and the brain's clearance response across the structural connectome. The ESM reproduces advanced Aß deposition patterns in the human brain (explaining 46∼56% of the variance in regional Aß loads, in 733 subjects from the ADNI database). Furthermore, this model strongly supports a) the leading role of Aß clearance deficiency and early Aß onset age during Alzheimer's disease progression, b) that effective anatomical distance from Aß outbreak region explains regional Aß arrival time and Aß deposition likelihood, c) the multi-factorial impact of APOE e4 genotype, gender and educational level on lifetime intra-brain Aß propagation, and d) the modulatory impact of Aß propagation history on tau proteins concentrations, supporting the hypothesis of an interrelated pathway between Aß pathophysiology and tauopathy. To our knowledge, the ESM is the first computational model highlighting the direct link between structural brain networks, production/clearance of pathogenic proteins and associated intercellular transfer mechanisms, individual genetic/demographic properties and clinical states in health and disease. In sum, the proposed ESM constitutes a promising framework to clarify intra-brain region to region transference mechanisms associated with aging and neurodegenerative disorders.

## Author Summary

Misfolded proteins (MP) mechanisms are a characteristic pathogenic feature of most prevalent human neurodegenerative diseases, such as Alzheimer's disease (AD). Characterizing the mechanisms underlying intra-brain MP propagation and deposition still constitutes a major challenge. Here, we hypothesize that these complex mechanisms can be accurately described by epidemic spreading-like interactions between infectious-like agents (MP) and the brain's MP clearance response, which are constrained by the brain's connectional architecture. Consequently, we have developed a stochastic epidemic spreading model (ESM) of MP propagation/deposition that allows for reconstructing individual lifetime histories of intra-brain MP propagation, and the subsequent analysis of factors that promote propagation/deposition (e.g., MP production and clearance). Using 733 individual PET Amyloid-ß (Aß) datasets, we show that ESM explains advanced Aß deposition patterns in healthy and diseased (AD) brains. More importantly, it offers new avenues for our understanding of the mechanisms underlying MP mediated disorders. For instance, the results strongly support the growing body of evidence suggesting the leading role of a reduced Aβ clearance on AD progression and the modulatory impact of Aß mechanisms on tau proteins concentrations, which could imply a turning point for associated therapeutic mitigation strategies.

## Introduction

Misfolded proteins (MP) are associated with aging processes and several human neurodegenerative diseases [1]–[3]. The prion-like hypothesis explains the neurodegenerative progression by the intercellular transfer of pathogenic proteins [4]–[6], under the perspective that MP behave like infectious-like agents that propagate from a few initial host regions to other brain regions. For instance, in Alzheimer's disease (AD), soluble Amyloid-ß (sAß) oligomers are thought to be the principal seeds that carry the misfolding process from region to region, accelerating the production/deposition of new misfolded proteins [7]–[9] and thus contributing to drive the pathology to new areas of the brain [10], [11]. The associated Aß toxicity has a relevant impact on AD development and progression [12]–[18]. The cell-cell transference is possible because sAß oligomers are very small assemblies of MP, which can be absorbed by axonal processes and transported to cell bodies, causing cytotoxicity in the receiving cells [10], [11], [19]. Also, sAß oligomers that are immersed in the extracellular fluid are subjected to the principles of molecular diffusion processes in the brain, i.e. a highly anisotropic movement along the axis of nervous fibers [20]. Consequently, sAß propagation, and the subsequent Aß deposition and cytotoxicity effects, occurs mainly between anatomically interconnected areas or between neighboring neuronal cells [10], [11], [21], [22].

Neuropathologic evidence supports the idea that each neurodegenerative disorder is linked to the misfolding of a specific protein or group of proteins [5], [23]–[25]. Thus, the network degeneration hypothesis proposes that misfolded proteins mechanisms should present disease-specific anatomical patterns [26]–[29]. Two recent studies showed that specific functional and structural covariance subnetworks of the healthy brain are in correspondence with the spatially dissociable cortical atrophy patterns of five distinct dementia syndromes [27], [30]. The reported link between structural/functional brain connectivity patterns and neurodegenerative damage supports the network degeneration hypothesis. This also emphasizes the strategic importance of developing molecular pathological approaches capable of reproducing MP propagation, which might not only be conducive to a better understanding of MP spreading factors, but could also help to evaluate their contribution to disease progression in relation with other postulated pathological mechanisms (e.g. the neuronal activity dependent degeneration [31]–[33]).

In this context, a Network Diffusion Model of disease progression in dementia was proposed [34], where the pathogenic proteins propagation follows the regional concentration gradients under the spatial constraints defined by the brain's connectional anatomy. Consistent with their theoretical predictions, the authors found that specific anatomical sub-modules are in correspondence with characteristic cortical atrophy patterns in AD and behavioral frontal temporal dementia. However, the ability of this model to replicate real MP propagation/deposition patterns remained unexplored. A potential limitation of this model is that it does not consider possible defense mechanisms of the brain. Rather, the disease factors can accumulate gradually, without system resistance, while inducing cellular death and cortical atrophy. Conversely, immunologic brain responses have been demonstrated to combat MP accumulation [35]–[38]. For instance, Aß clearance by macrophages and microglia cells are responsible in part for the remarkable fluctuations in neurological functions that AD patients present even during the same day [26], [35], [39]. Furthermore, recent evidence indicates that initial Aß related processes could have a protective role on the nervous system [40], [41], which suggests non Aß related neurodegenerative effects (e.g. cellular death and cortical atrophy) at all the Aß propagation states but only after an abnormal accumulation process.

Considering the relevance that both intercellular MP transfer and associated clearance defenses have toward the development of neurodegenerative disorders, here we proposed a stochastic epidemic spreading model (ESM) to describe the dynamic interactions between MP infectious-like agents and the brain's clearance response. The validity/applicability of the proposed hypothesis and model was explored using 733 individual PET Aß datasets from the Alzheimer's Disease Neuroimaging Initiative (ADNI). We found that the ESM is able to reconstruct individual/group Aß deposition patterns. Most importantly, ESM predicts that it is not an increased Aß production but mainly a deficit in Aß clearance processes and an early Aß onset age that result in the formation of an excessive Aß deposition pattern, and in the conjectured acceleration of the preceding tauopathy. Additionally, our results highlight the strategic role of the MP outbreak regions and their connectional architecture on the disease's temporal progression, as well as the impact of individual genetic and demographic properties on intra-brain Aß propagation.

## Results

### Recovering the lifetime individual histories of Aß propagation/deposition

**Figure 1:**
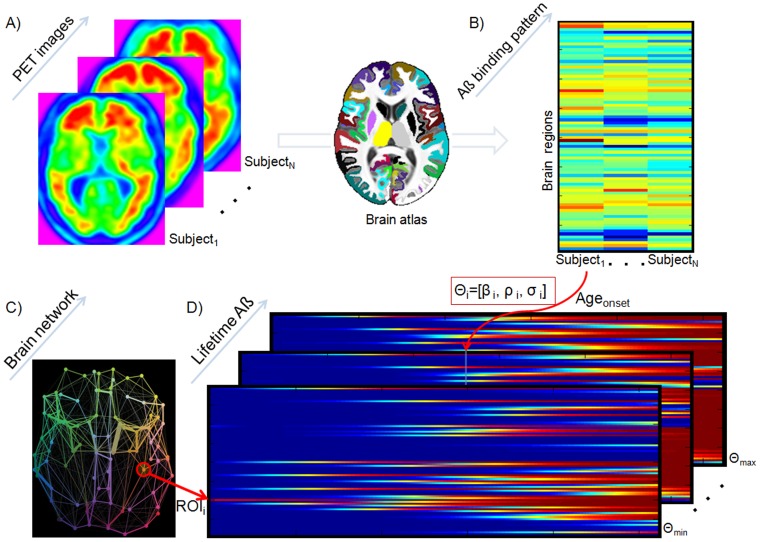
Reconstruction of individual Aß propagation/deposition histories using an ESM. ^18^F-AV-45 PET scans (**A**) are used to calculate individual Aß deposition patterns for different regions covering all the brain's gray matter (**B**). Then, detailed region-region anatomical connectivity information from a young healthy group (**C**) is used to generate multiple hypothetical lifetime Aß propagation/deposition courses (**D**). Each hypothetical course corresponds to an initial set *i* of sAß spreading seed regions and a different set of global model parameters 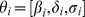. Then, a selective iterative algorithm estimated, for each subject, the model parameters that maximized the similarity between the generated and the reference Aß deposition pattern, as well as the time point at which this maximization occurred. The latter output was used to calculate the individual onset age of Aß binding, which in conjunction with the obtained model parameters were assumed to characterize each subject's Aß propagation/deposition history.

We developed a stochastic epidemic spreading model (ESM) to describe intra-brain Aß propagation and deposition processes (*Methods* section). Then, we proceeded to explore the ability of the model to reproduce Aß deposition patterns in healthy and pathological brains. Figure 1 illustrates the key processing steps of our approach. First, we used Florbetapir (^18^F-AV-45) PET data to quantify Aß deposition patterns in a cohort of 733 subjects with non-Hispanic Caucasian ancestry (Table S1) from the ADNI database (*Methods, Study participants, Dataset 1*). Each participant was previously diagnosed as healthy control (HC, n = 193), early mild cognitive impairment (EMCI, n = 233), late mild cognitive impairment (LMCI, n = 196) or probable AD (n = 111). For each subject, the baseline ^18^F-AV-45 PET scan was employed to calculate the Aß deposition probabilities for 78 regions covering all the gray matter [42], and these were used to define the individual Aß deposition pattern (*Methods, Regional Aß deposition patterns*). Next, we used the developed ESM, and region-region anatomical connectivity information from 60 healthy young subjects (*Methods, Study participants, Dataset 2*), to generate multiple hypothetical regional courses of Aß propagation/deposition. Each hypothetical generation corresponded to a specific set of sAß spreading seed regions, up to a maximum of 6 regions consisting of all possible combinations, and a set of model parameters from which we simulated 50 years of propagation starting with Aß presence only in the seeds. A selective iterative algorithm (*Methods, Model exploration/validation*) was used to identify the seed regions that better explained the PET-based Aß deposition patterns across the study cohort, as well as the individualized model parameters that maximized the similarity between the generated and the individual reference Aß deposition patterns. In sum, a set of the most likely Aß outbreak regions were identified, assuming the same set of regions for the whole sample, whereas for each subject four different model parameters were estimated: Aß production rate (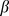), Aß clearance rate (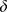), onset age of Aß outbreak (Age_onset_), and model noise level (σ). For further details see *Methods (Model exploration/validation* subsection) and Figure 1.

**Figure 2:**
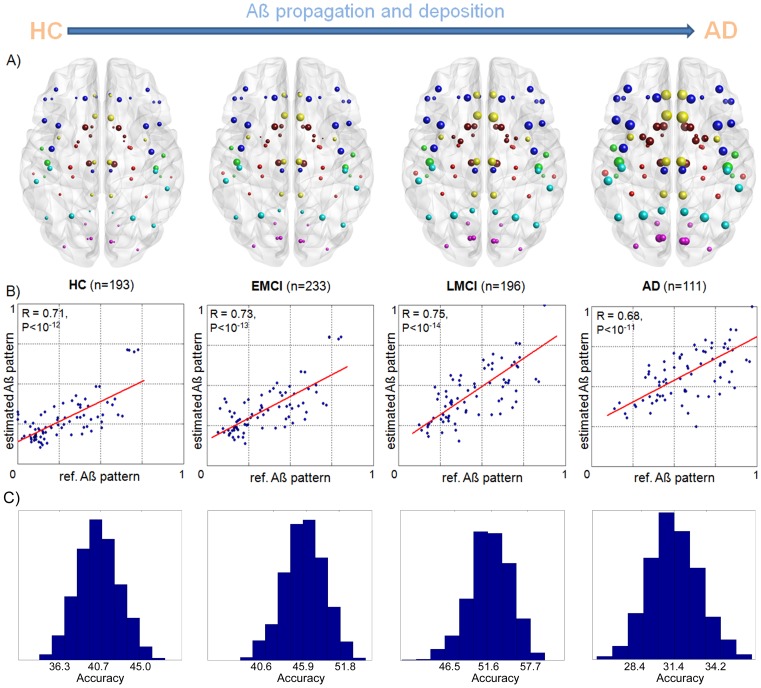
Characteristic regional Aß deposition patterns in healthy and pathologic brains. **A**) PET-based mean regional Aß deposition probabilities (adjusted by age, gender, and educational level) in HC, EMCI, LMCI and AD groups. Nodes correspond to 78 regions covering all the brain's gray matter, with node sizes proportional to the associated Aß burden. Note the progressive expansion of the Aß deposition, starting mainly from DMN regions to the rest of the brain. This supports the development of an abnormal Aß deposition pattern in correspondence with the disease progression (from HC to advanced AD clinical states). **B**) Correspondence between the estimated and PET-based mean regional Aß deposition probabilities for the different groups. **C**) Prediction accuracy distributions obtained for the different groups (via a repeated random sub-sampling cross-validation procedure).

Consistent with the hypothesis of an intra-brain Aß epidemic spreading behavior, our propagation/deposition model reproduced, from the remote non-binding states, the characteristic Aß deposition patterns in the adult cohort (Figures 2A,B). It explained between 46.4∼56.8% (all P<10^−10^) of the variance in mean regional Aß deposition probabilities (adjusted by age, gender, and educational level) in HC, EMCI, LMCI and AD groups. See Table S2 for a comparison with previous approaches. In addition, it identified the posterior and anterior cingulate cortices as the most probable starting seed regions for the Aß propagation process (see Table S3 for examples of other tested combinations of regions, based on previous reports). The cingulate cortex, particularly its posterior area, is considered a core node of the default mode network (DMN), and is thought to be involved in self-relevant/affective decisions, mental simulation, and integration tasks [43], [44]. This result is in agreement with the large amount of evidence suggesting the critical role of the DMN on the genesis and propagation of AD [31], [45], [46]. For a complementary seeds identification analysis, see *Discussion section* (*Identification of the MP propagation epicenter* subsection) and Figure S1.

Next, we re-evaluated the competence of the ESM framework to reproduce prion-like spreading mechanisms, but now based on the idea that, if the ESM is describing real intra-brain propagation of MP, then alterations in the structural connectional information should affect the model's results negatively. We tested this by comparing the capability of the ESM to explain advanced Aß deposition patterns, using the available connectivity information and alternatively using “non-informative” connectional information. For this, 100 randomized versions of the original anatomical connectivity matrix were created (preserving its weight, degree and strength distributions [47]), and the propagation model was evaluated for each of these versions. We observed a significantly higher model competence (all P<10^−5^) to explain the Aß deposition patterns when the original anatomical connectional information was used (Table S4). This result supports the ability of the SEM to describe real MP spreading processes, based on the central interrelation between biological factors directly related to these pathogenic proteins (e.g. Aß production and clearance) and the complex connectional architecture of the human brain.

Finally, the statistical robustness and predictive power of the introduced ESM was tested via a repeated random sub-sampling cross-validation. Each clinical group (HC, EMCI, LMCI and AD) was randomly split into training and test data of the same size. For each such split, the model values derived at the group level for the training data were used to test the predictive validity of the model on the validation group. We observed significant predictive power across the different clinical states (Figure 2C), with prediction accuracy values ranging from 40.7% (95% CI: 36.3, 45.0) for the HC group to 31.4 (95% CI: 28.4, 34.2) for the AD group (Table S5). Slightly lower prediction accuracy was observed for the AD group. We attribute this to the smaller sample size, in comparison with the other groups, and as will be analyzed in the next subsections, to a larger period of Aß propagation/deposition processes (with a significantly earlier propagation onset). This larger period of the phenomenon to be modeled can be consequently associated to a larger accumulation of model errors.

### Predicting regional Aß arrival time with effective anatomical distance to outbreak region and connectivity degree

Historically, the identification of outbreak nodes has been considered a primary step towards the spatiotemporal understanding of epidemic phenomena [48]. In the context of brain neurodegenerative disorders, functional proximity to epicenter regions implies greater disease-related regional vulnerability [30]. This suggests an organized pattern for propagation of disease agents, in accordance with the trans-neuronal network-based MP spread hypothesis, and supports the key role of specific epicenter regions in the disease progression processes. Those results were obtained using an indirect measure of MP presence, i.e. gray matter atrophy quantified using voxel-based morphometry. However, the relation between gray matter atrophy and MP effects is still unclear, and, in addition, the former can also be caused by multiple different factors (e.g., vascular dysregulation).

**Figure 3:**
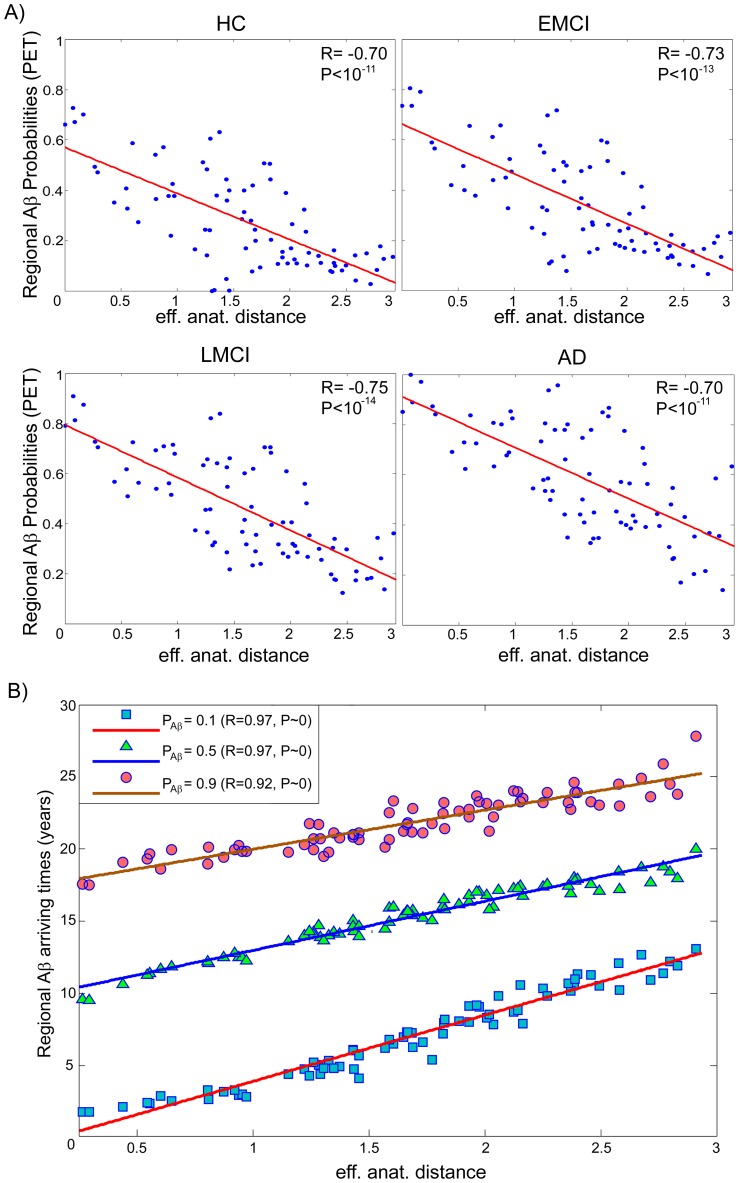
Effective anatomical distance to outbreak regions modulates the Aß propagation processes. A) PET-based regional Aß deposition probabilities for the different groups vs effective anatomical distances. B) Regional Aß arriving times vs effective anatomical distances, for different Aß probability thresholds (i.e. 0.1, 0.5 and 0.9). In A) and B), note the co-linearity between different clinical states or Aß probability thresholds, with more advanced disease states corresponding to higher depositions and propagation times. See also Figure S2.

To obtain direct evidence of MP dispersion as a function of proximity to an epicenter, we first explored the relation between the PET-based regional Aß deposition patterns and the effective anatomical distances to the identified Aß outbreak regions (anterior and posterior cingulate cortices; *Methods, Model exploration/validation*). We observed a significant negative linear relationship between these two variables, across the four clinical states (Figure 3A). Interestingly, best-fit lines for the different clinical groups displayed a consistent co-linearity (Figure 3A and Figure S2). The relationships were characterized by similar slope but different Aß deposition intercepts that increase according to disease progression. We verified that these associations are not explainable by the spatial proximity between regions (Table S6). These results support the role of the outbreak regions as centers of radial disease factor propagation, which is modulated by the brain's connectional architecture.

Next, we used the spatiotemporal information provided by the ESM to analyze the link between regional Aß arriving times (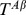) and the effective anatomical distances. For each brain region *i*, 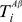 was calculated as the time at which the Aß probability deposition of this region reached a given threshold (e.g. 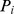 <0.9 implying no deposition, 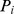 ≥0.9 implying deposition). In line with the previous results, we found a significant linear predictive relationship between the effective anatomical distances and the 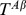 values (Figure 3B). The shape of this relationship was invariant to the selection of different Aß deposition thresholds.

Notably, these results correspond with the linear predictive relationship reported for effective distances in human social networks and disease arrival times for real epidemics propagation data [49] (e.g. 2009 H1N1 pandemic). This parallelism between intra-brain MP propagation mechanisms and epidemic propagation in human disease networks [49], supports our hypothesis of an intra-brain epidemic spreading behavior of MP propagation. Furthermore, these model-based findings clarify the distance-vulnerability effects observed for gray matter atrophy [30] and Aß deposition (Figure 3A).

In terms of regional vulnerability to disease pathological effects, recent studies have also suggested a direct link between structural/functionally connectivity levels and regional vulnerabilities [31], [50], [51]. Highly connected brain regions are usually known as “hubs nodes” of the brain network (for review see [50]). Buckner et al., 2009, showed a high correspondence between Aß deposition levels and functional connectivity in the brain hubs. Further evidence, based on meta-analyses of published magnetic resonance imaging data about 26 different brain disorders, suggest that pathological brain lesions (i.e. gray matter atrophy lesions) are mainly concentrated in structural hub regions, independently of the studied disorder [51]. This fact is considered a consequence of the high topological centrality and biological cost of the hubs, which make them more vulnerable to a diverse range of pathogenic processes [31], [51]. In order to explore if the introduced ESM can clarify this connectional-pathogenic association, we analysed the relation between regional anatomical connectivity degrees and Aß arrival times, as measures of "hubness" (*Methods*, *Anatomical connection probability*) and temporal vulnerability to receive aberrant disease factors, respectively. We observed significant negative correlations (all P<10^−9^) between these two variables, independently of the selection of different Aß deposition thresholds (see Figure S3). This suggests that regions with higher anatomical connectivity degrees experience early Aß arrival and, consequently, larger periods of exposition to the negative effects of this aberrant protein.

### A malfunctioning Aß clearance system and an early Aß onset age are major factors associated with AD

For decades, Aß propagation and accumulation has been thought to have a causal role on the cascade of cognitive/clinical events leading to AD [52], [53]. For instance, Aß toxicity has been causally associated with brain oxidative stress [14], [18], mitochondrial dysfunction [18], synapse and spine loss [13], widespread neuronal dysfunction and cell death [12], synaptic plasticity and memory impairment [16], [17]. To test the potential clinical impact that progressive Aß presence can have on the pathology's progression, we studied whether model variables controlling intra-brain Aß propagation/deposition are related to AD and intermediate cognitive/clinical states. For this, we considered the clinical diagnosis (HC, EMCI, LMCI or AD) as a dependent variable in a Multinomial Logistic Regression model with Aß production/clearance rates, noise and onset age as independent variables (controlling by gender, age and educational level). Then, the contribution of each regressor was evaluated using a robust metric of relative importance in prediction analysis (*Methods, Statistical Analysis*).

**Figure 4:**
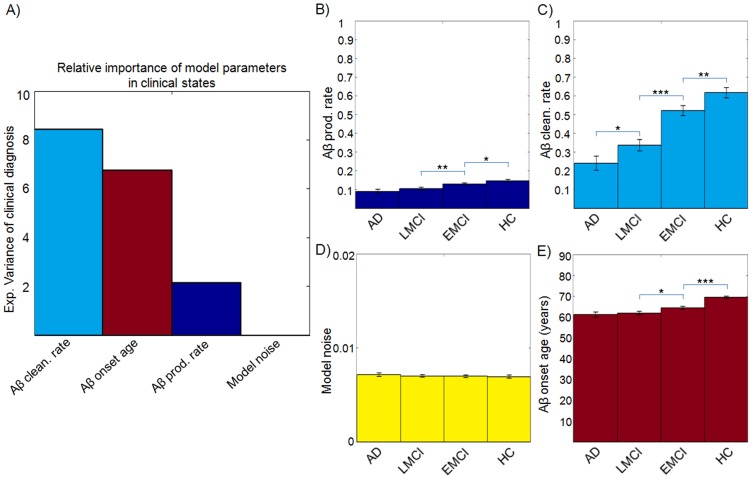
Subjects with different clinical states presented different Aß propagation histories. **A**) Explained variance of the clinical diagnoses (HC, EMCI, LMCI and AD) by the different Aß propagation model parameters. Mean (± standard error) Aß production rate (**B**), Aß clearance rate (**C**), noise standard deviation (**D**) and onset age of Aß propagation (**E**) for the different clinical groups (adjusted for gender and educational level). *p<0.05, **p<0.01, ***p<10^−5^, Student's *t*-test.

We observed a statistically significant relationship between clinical diagnosis and Aß clearance rate and onset age (Figure 4 and Table S7). The clearance rate was found more related to the clinical diagnosis than the other model parameters (Figure 4A), explaining 8.45% (95% CI: 4.88, 11.89) of its inter-subject variance. A closer look at the differences between the four clinical groups (Figures 4B-E and Table S8), revealed that the clearance rate is also the model variable with the most consistent variance across the different clinical diagnoses. It is followed by the onset age of Aß accumulation, which reflects a decreasing transition from HC to EMCI-AD states (Figure 4D) and explains 6.77% (95% CI: 3.42, 9.88) of the variance in clinical diagnoses. We observed a significant decreasing trend on the Aß production rate from HC to EMCI-AD states (Figure 4B), however the impact of this effect on the clinical diagnosis was not significant (95% CI: −0.76, 3.77; for further analysis, see Text S1). While the individualized global Aß production rate can be seen as a measure of the lifetime individual regional potential to produce Aß infectious-like factors, the corresponding clearance rate reflects the lifetime intrinsic capacity to combat the Aß accumulation. Therefore, these results suggest that although a significantly earlier onset age of Aß accumulation and a non-significantly lower production rate of Aß agents are associated to AD, a deficiency associated to Aß clearance might be the most determining Aß-mediated factor for the development of the disease.

### APOE e4 genotype multi-factorial impact on Aß propagation/deposition

Apolipoprotein E (APOE) e4 genotype is considered a relevant genetic risk factor for AD and intermediate MCI states [54]. It has been associated to Aß aggregation into fibrils [55], the hindered clearance of sAß [56], and neurodegeneration by directing toxic Aß oligomers to synapses [54], [57]. Using our ESM, we explored how different APOE e4 genotypes impact Aß propagation and deposition. For each model parameter, we performed a three-way ANOVA test considering as grouping parameters the number of APOE e4 allele copies, as well as the gender and educational level of the participants.

**Figure 5:**
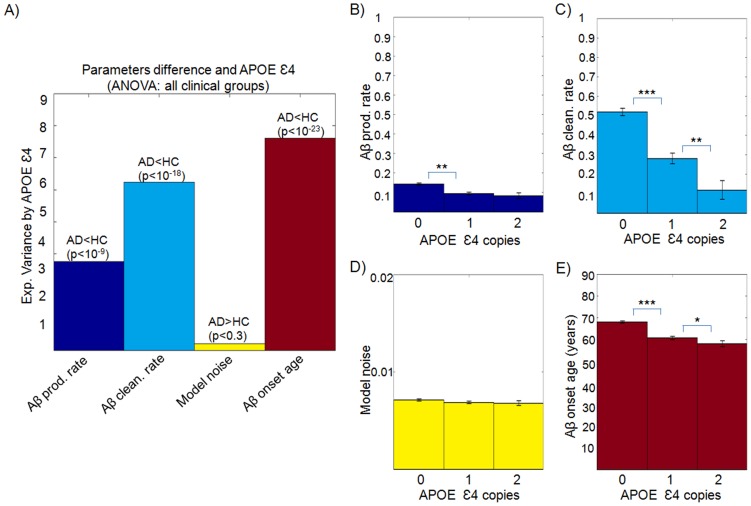
Multi-factorial impact of APOE e4 genotype on Aß propagation/deposition. **A**) Explained variance of the propagation model parameters by the different APOE e4 genotypes (zero, one or two e4 allele copies). Mean (± standard error) Aß production rate (**B**), Aß clearance rate (**C**), noise standard deviation (**D**) and onset age of Aß propagation (**E**) for the different number of e4 allele copies (adjusted for gender and educational level). *p<0.05, **p<0.01, ***p<10^−10^, Student's t-test.

We observed a significant effect of APOE e4 genotype on the Aß production/clearance rates and on the onset age (Figure 5A and Table S9). In particular, we found that APOE e4 genotype had highest impact on the onset age, decreasing it proportionally to the number of APOE e4 allele copies (Figures 5A,E), and explaining 13.21% of its inter-subject variance (P = 1.12×10^−24^, F = 59.57). This result is in line with previous reports associating APOE e4 genotype with an earlier age at disease onset and a faster AD pathological progression [58], [59]. In addition, we observed a significant decrease in Aß clearance rate with regard to the number of APOE e4 allele copies (Figures 5A,C), explaining 10.48% of its inter-subject variance (P = 2.24×10^−19^, F = 45.60). This supports our previous result associating AD onset with an Aß clearance deficiency and, more importantly, evidences that this clearance deficiency partly has a genetic cause [56]. We also found significant effects of APOE e4 on Aß production rate (Figures 5A,B; P = 5.38×10^−19^, F = 21.98), which reflects the multi-factorial influence of this genotype on the evolution of AD and intermediate MCI states [54]–[57].

Further statistical analyses were performed to assess how the specific number of APOE e4 allele copies impact on Aß propagation and deposition (Table S10). We found that the effects due to the presence of two e4 allele copies are more relevant (in terms of the model parameters) than the effects due to the presence of only one copy (Figures 5B–E and Table S10). This is in agreement with the reported semi dominant inheritance effect of APOE genotype on developing AD [60].

When investigating the relationship of the model parameters with the demographic variables, we also found a significant impact of gender on Aß production rate (P = 1.90×10^−3^,F = 9.68), Aß clearance rate (P = 1.35×10^−3^,F = 19.19) and Aß onset age (P = 2.06×10^−3^,F = 36.84) (Tables S9 and S11). For all these cases, female gender was associated with significantly lower model parameter values (Table S11). This result is in high correspondence with the fact that women are more likely to develop AD than men [61], [62]. Furthermore, we found a significant interaction between APOE e4 genotype and gender, which together are modulating the Aß onset age (P = 1×10^−5^, F = 9.30). This is consistent with the higher propensity for women to develop AD across most ages and APOE genotypes [62], with the most pronounced detrimental effect of APOE e4 on DMN connectivity and CSF tau levels [61], and with the reported greatest amyloid plaque and neurofibrillary tangle pathology for women [63].

Finally, when investigating the relationship of the noise parameter σ with APOE e4 and the demographic variables (Table S9) we found that female subjects with a higher educational level have a higher noise level (P = 0.019, F = 5.45). In conjunction with a significant impact of gender and educational level on the Aß onset age (P = 0.01, F = 5.45) and a non-significant trend effect of educational level on Aß clearance rate (P = 0.093, F = 2.82), this may be reflecting the complex relationship that exists between Aß binding, gender, cognitive reserve and clinical state [64]. The larger variability in Aß deposition patterns associated with higher noise, gender and educational level, could explain the disputed results of the cognitive reserve hypothesis [65]–[67].

### Modulatory impact of Aß propagation/deposition history on CSF Aß^1-42^, t-tau and p-tau levels

CSF measures of Aß, total tau (t-tau) and phosphorylated tau (p-tau) are considered the most relevant early biomarkers of AD [68], [69]. Although Aß and tau proteins were historically considered to arise and act independently, now it is thought that both proteins are strongly interrelated [13]. Based on different converging evidences, it has been suggested that Aß pathophysiology might drive and accelerate pre-existing tauopathy [70]. Here, we aimed to re-evaluate this interrelation hypothesis under the assumption that, if the intra-brain ESM of Aß propagation/deposition can reflect Aß pathophysiology accurately, then abnormalities in CSF Aß, t-tau and p-tau concentrations should be correctly reflected by the individualized model parameters. For this, we used CSF Aß^1-42^, t-tau and p-tau^181^ measures from a subsample of 307 healthy and non-healthy subjects belonging to the ^18^F-AV-45 PET scanned group (*Methods, CSF measures*). For each CSF measure, we performed a seven-way ANOVA test, considering the model parameters, age, sex and educational level as modulatory factors.

**Figure 6:**
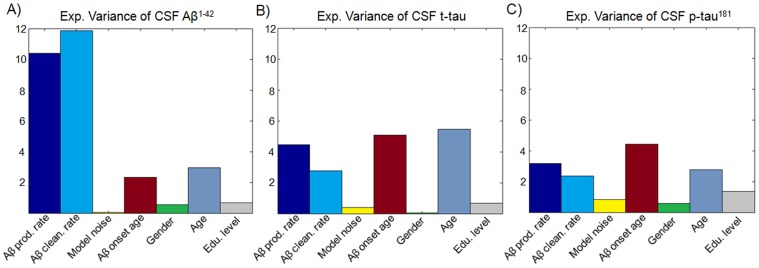
Influence of parameters controlling Aß propagation/deposition on CSF Aß^1-42^, t-tau and p-tau^181^ levels. While CSF Aß^1-42^ (**A**) is mainly influenced by Aß production/clearance rates, t-tau (**B**) and p-tau (**C**) are highly influenced by chronological age and Aß onset age (combined, these two temporal factors should reflect the interrelation period between amyloid pathophysiology and tauopathy).

The results (Figure 6A and Table S12) show a significant impact of Aß production/clearance rates on CSF Aß^1-42^, explaining 10.40% (P = 1.24×10^−12^, F = 55.02) and 11.85% (P = 4.83×10^−14^, F = 62.66) respectively, of its across-subject variance (see also Text S1). We also found that the Aß onset age and the chronological age are significant modulators of CSF Aß^1-42^, explaining 2.31% (P = 5.36×10^−4^, F = 12.24) and 2.97% (P = 9.29×10^−5^, F = 15.69) respectively, of its variance. Together, all considered modulators accounted for 28.82% of the CSF Aß^1-42^ variance. Aß production/clearance rates were also found to have significant impact on CSF t-tau, explaining 4.45% (P = 1.68×10^−5^, F = 19.33) and 2.77% (P = 6.32×10^−4^, F = 11.93) respectively of its variance. However, in this case the higher impacts correspond to the Aß onset age and chronological age (Figure 6B), with 5.08% (P = 4.41×10^−6^, F = 21.87) and 5.45% (P = 2.07×10^−6^, F = 23.44) respectively, of explained variances. Similar effects were observed for CSF p-tau (Figure 6C), for which Aß onset age was the strongest modulator and accounted for 4.44% (P = 2.07×10^−5^, F = 18.23) of its variance.

According to these results, while Aß production/clearance rates might be influencing the deposition and recirculation of Aß and subsequently its inter-relationship with tau proteins, the observed Aß onset age and chronological age effects on t-tau and p-tau may be reflecting the time duration of such inter-relationship. These results are consistent with the idea of an interrelated pathway between amyloid pathophysiology and tauopathy [70], [71] and, in combination with results from the previous subsections, they are also consistent with the notion of an associated failure to clear mislfolded proteins [70], [72].

## Discussion

Characterizing the mechanisms underlying intra-brain MP propagation and deposition constitutes a major challenge of the molecular pathological approaches devoted to the study of neurodegenerative disorders. Here, we showed that these complex mechanisms can be biophysically described by epidemic spreading-like interactions between the infectious-like agents (misfolded proteins) and the brain's clearance response, across the human structural connectome. We identified several genetic, structural and demographic factors associated to the biophysical model variables controlling these interactions. The proposed ESM constitutes a promising framework to clarify intra-brain region to region transference mechanisms related to aging and neurodegenerative disorders.

### The prion-like hypothesis and the misfolded proteins epidemic spreading behavior

The prion-like hypothesis explains the neurodegenerative progression by the intercellular transfer of pathogenic factors [4], [73]. This perspective presents a striking similarity with the spread of real infectious diseases in biological populations. Social networks constitute a common structural substrate over which infectious factors propagate, reaching in some cases an epidemic/uncontrollable behavior [74]. Independently of the pathogenic agent's characteristics, its propagation dynamics are always constrained by the connectivity structure of the attacked system. It is in this context that we hypothesized the Aß proteins propagation and deposition as a natural epidemic spreading event, whose dynamics are determined by infectious-like agents and immunologic response actions that compete under a restrictive anatomical network (the structural human connectome). Note, however, that the term *infectious* does not necessarily imply the presence of fully negative propagating factors, since the genesis and role of MP in the brain are not completely understood [40].

Previous studies have used the brain's structural and functional connectivity to explain neurodegenerative atrophy patterns (for recent reviews see [75], [76]). We extended previous connectivity-based approaches [27], [30], [34] by combining pathogenic factors actions (production and spreading) with possible defense responses, including also the influence of stochastic or undetermined processes. The inclusion of basic biological variables (e.g. MP production/clearance rates, time of propagation) provides a more realistic characterization and understanding of the studied phenomenon, allowing not only to reproduce the MP dynamics but also to identify the genetic, structural, and demographic factors associated to it. For purposes of comparing different methods, we applied the Network Diffusion Model (NDM) [34] to the same Aß datasets and connectivity information (for further details see Table S2). We found that NDM also identified the posterior and anterior cingulate cortices as the most probable starting seed regions for the Aß propagation process. However, even when the obtained mean regional explained variance for the NDM was around 27–33%, with a significant statistical association (p<0.05), the corresponding root mean square errors (RMSEs) were considerably high, reflecting large absolute differences between estimated and reference Aß concentration patterns. In addition, Akaike Information Criterion (AIC) values evaluated for both models (ESM and NDM) revealed a significantly lower accuracy performance for the NDM (P = 7.13×10^−8^, Z = −5.26), independently of the number of models parameters. We noted that although the NDM is capable of dispersing the initial infectious-like factors from the seed regions to the rest of the brain network, such dispersion is at the expense of the local concentrations, which after the initial exchange decreases continuously. As a consequence, the total Aß concentration is never higher than the “injected” amount and after a given time the propagation of the factors stops. This behavior is not physiologically realistic as shown in the literature [77]. Note that this issue is a consequence of the absence of a source term in the NDM, which is included in the ESM.

In addition, consistent with reported associations between functional proximities to a pathogenic epicenter and gray matter atrophy levels [27], [30], we found that effective anatomical distances to the Aß outbreak regions can predict regional Aß depositions and arrival times values. In terms of prediction accuracy, anatomical connectional proximities to the epicenter seem to be more interrelated to Aß levels than functional proximities to gray matter atrophy levels (Table S2). This might be responding to several possible causes, such as: a) a tentative higher impact of the anatomical connectivity (implying only direct links) than the functional connectivity (implying both direct and indirect links) on pathogenic agents propagation, b) the use on [27], [30] of indirect measures of MP presence to evaluate prion-like mechanisms, i.e. gray matter atrophy quantified using voxel-based morphometry, and c) the fact that gray matter atrophy can be caused by multiple pathogenic factors (e.g., vascular and metabolic dysregulations). In addition, in these studies the nodes of the analyzed networks were obtained based on a priori statistical selection of the significantly affected brain regions in the diseased group, ignoring other brain regions, which may have introduced a bias in the posterior atrophy level vs functional proximity analysis.

### Anatomic connectivity impact on intra-brain MP propagation

The intercellular transference of pathogenic proteins (e.g. across axonal projections [10], [19], or the extracellular space that is constrained by the connectional architecture [20]), is a major statement of the prion-like hypothesis. The ability of ESM to reconstruct Aß deposition patterns from early to advanced disease states, suggest the methodological importance of considering the structural connectional information on the modeling of MP propagation/deposition mechanisms. However, these alone do not offer an evaluation of the real contributions/advantages of using or not the connectional information on MP propagation modeling. We tested this contribution by comparing the ability of the introduced ESM framework to explain advanced Aß deposition patterns, using the available connectivity information and alternatively using equivalent non-informative connectional information (Results, first subsection, and Table S4). The results supported a significantly higher model competence to explain Aß deposition patterns when the original anatomical connectional information was used. Similarly, effective anatomical distances estimated using the structural human connectome were significantly better predictors of regional Aß levels (all P<10^−11^) than the equivalent distances estimated using the randomized networks. In addition, we verified that spatial proximities of the considered 78 regions to the identified epicenter (cingulate cortex) cannot explain the observed predictive relationship between effective anatomical distances to epicenter and regional Aß levels (Table S6). Together, these results add evidence to the previously postulated prion-like mechanisms associated to Aß proteins [10], [19], [39] and, furthermore, highlight the methodological importance of considering structural brain connectivity information for the study of such processes.

In accordance with previous studies [31], [32], [50], [51], our results support a significant relationship between regional connectional degrees and pathological vulnerability. We observed that regions with higher connectivity degrees are primarily targeted by the Aß factors (Figure S3). This suggests that hub regions in the brain are most extensively exposed to the negative effects of these aberrant proteins. Considering that similar connectivity-based modulatory effects could be presented for different MP (e.g. tau, α-synuclein) or even, as previous literature is suggesting, for other transferable region-region pathogenic mechanisms (e.g. metabolic or functional dysregulations), this phenomenological relationship could explain the spatiotemporal association between regional hubness and lesional levels observed for different diseases.

### Identification of MP propagation epicenters

Converging evidence suggests that cingulate cortex is one of the earlier structures presenting Aß deposition [29], [31] and other structural/functional alterations related to AD progression [31], [46], [51]. Therefore it is not surprising to find this structure as a strong candidate to be the Aß outbreak region and to have a consistently high Aß deposition across the whole sample. However, with the current data it is hard to determine whether the identification of this structure, or any other, as propagator epicenter is more related to: a) the fact that it is the real Aß outbreak center, or b) it is located spatially close to the real outbreak, or c) its selection is reflecting a model limitation (e.g. potentially, the model could not identify peripheral regions but only some kind of “best MP propagator”). Despite this intrinsic limitation, we know that while a realistic selection should be influenced by the deposition level of each seed candidate, it should also depend on the seed's anatomical connectivity patterns. In order to explore this and its relation with our previous results, we performed a complementary analysis for the seed identification, now assuming that the regional connectional proximities to the real outbreak should be capable to reflect the regional levels of Aß deposition, as a direct consequence of an aberrant factor propagation from the initial center [49]. For this, we calculated the correlation between the regional Aß deposition values and the effective anatomical distances to each considered brain region. The results (Figure S1) suggested that, in accordance with the previous reasoning, the cingulate regions are the most likely candidates to have a leading role in Aß outbreak and subsequent propagation, presenting the highest correspondences between connectional distances and brain Aß deposition levels. However, a conclusive validation of these results requires more integrated data, e.g. longitudinal PET Aß datasets from initial to more advanced Aß binding states, and/or animal models to evaluate the level of mismatches between a given MP/Aß injection site and the corresponding identified epicenter.

### Aß clearance deficiency and implications for medication therapies

Historically, Aβ binding features associated with AD have been assumed to be a causal consequence of the imbalance between Aβ production and clearance. With the exception of rare genetic forms of AD [78], an increased Aß production in typical AD patients has not been consistently confirmed [38], [79]. In fact, recent metabolic labeling analyses in a cohort of AD (n = 12) and normal controls (n = 12) revealed a non-significant inter-group difference in the Aβ production rates, while clearance rates were found significantly decreased in the AD group [38]. Moreover, new evidence suggests that under certain conditions Aß proteins may play a protective role on the nervous system [40], [41]. Our results are in agreement with a decreased Aß clearance capacity associated with AD onset [38], [39]. Once we evaluated the ESM variables associated to Aß propagation/deposition with real data, we found a significant association between individual Aß clearance rates and clinical states. In terms of the relative importance as a clinical predictor, the clearance rate demonstrated a considerably higher impact in comparison with the other variables (i.e. Aß onset time and production, physiological/external noise). This result strongly suggests that differences in the capacity to clear/degrade the Aß proteins result in vast differences in the net amounts ending up in the receiving cells [39], which can cause more seeding and pathology in these cells. At the cellular level, ubiquitin–proteasome and the autophagy–lysosome pathways are considered the two main routes for intracellular MP protein degradation [72]. Although the ESM cannot distinguish between these two pathways, the obtained results are in some way highlighting the importance of considering these and other similar mechanisms related to MP clearance, which might have a relevant role on the development and progression of different neurodegenerative diseases. Specifically, the growing body of evidence supporting a reduced Aβ clearance in AD development could imply a turning point for associated therapeutic mitigation strategies [35], [39], [72]. A significant increment of Aβ and tau clearance capacities attending in parallel to the individual genotype and demographic characteristics, through an immunologic reinforcement [35], [80], or through a genetically induced enhancement [81], may be, tentatively, an alternative to combat AD onset and progression, with the subsequent impact on the associated undesirable symptoms.

### Aβ and other factors contributing to neurodegeneration

Although ESM was created to describe the spatiotemporal propagation of different MP (Aß, tau, α-synuclein, superoxide dismutades-1, etc), here it was explored only with Aß patterns. As it is well known, Aß is not the unique pathogenic factor associated with neurodegenerative progression. In AD and intermediate mild cognitive states, tau proteins are thought to also have a primary role on disease progression, presenting a higher correspondence than Aß to gray matter atrophy patterns and clinical states [70]. However, Aß toxicity has been evidenced in different forms and its negative role is currently the subject of scientific debate. Aß toxicity has been causally related to brain oxidative stress [14], [18], mitochondrial dysfunction [18], synapse and spine loss [13], widespread neuronal dysfunction and cell death [12], synaptic plasticity and memory impairment [16], [17]. Moreover, as evidenced by previous studies [13]–[15] and as was verified in our analysis (Results, Figure 6), Aß proteins have a significant modulatory impact on tau proteins concentrations.

A hierarchical model of Aß acting on tau is supported by several lines of evidence [13]. As a consequence, many scientific groups are now emphasizing the strategic importance of considering the mutual interrelation (Aß, tau) as an alternative to get a better understanding of the pathogenic mechanisms and clinical consequences associated to both proteins [80]. Furthermore, the current consensus is that a given clinical state can not only be caused independently by one protein (Aß or tau), but it can also derive from complex interactions between these and/or other contributing factors (e.g. metabolic, vascular and functional dysregulations).

### Methodological issues and future work

This study presents several limitations. The anatomical connectivity information used in the model evaluation still presents notable limitations [75], [82]. In this sense, current difficulties to distinguish between real and spurious connections may have a significant impact on the results, by distorting the modeled structural relations among the regions and consequently the probabilities of receiving or not new MP agents from connected regions. The symmetrical property of the connectivity matrix, which is the result of the limitations of current diffusion MRI tractography techniques, makes it impossible to consider anterograde or retrograde MP propagation processes, which could potentially present different kinetic mechanisms. Additionally, the use of a gray matter parcellation template with variably sized regions [42], may introduce a significant bias in the connectivity evaluation. The probabilistic connectivity measure used in this study is based on regional maximum-voxel levels of connectivity, and thus reflects the fibers orientational coherence across the estimated connecting fibers pathways [83]. This approach may be less sensitive to the sizes of the regions than other traditional probabilistic connectivity measures, e.g. the frequentist ratio between the number of connecting and generated fiber pathways, with different seed sizes implying different number of generated paths. However, independently of the connectivity approach used, it is still not clear how to evade the influence of the nodes selection on the structural network's estimation [84]. While model estimation at the voxel level could be a tentative solution to this issue, such approach would imply other limitations, such as low inter-subjects correspondence across nodes, the difficulty to interpret regional findings, and a considerably high computational cost. In the ESM, regional MP production and clearance rates depend of the local MP deposition probabilities, which implies different effective rates across the time and brain regions. But, at the same time, these local effective rates are subjected to global individual parameters of MP production and clearance (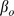 and 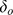 in Equations 4 and 5, respectively). This generalization may cause the loss of real variability at the local tissue levels, such as possible spatial differences in MP production/clearance rates due to changes in neuronal and glial cells properties across the cortex. However, the inclusion of regional production/clearance parameters in the model could lead to a very high dimensional parameters space (i.e. with at least two additional parameters per region), and subsequently result in considerably higher difficulty to evaluate these at individual levels. In addition to these limitations, errors in defining an appropriate gray matter parcellation scheme [85] and the characteristic low signal to noise ratio of the 18F-AV-45 PET data are factors that may affect the global capability of the proposed ESM to predicts regional Aß deposition patterns.

This study can be extended in multiple directions. These should include further validation of the developed formulation with animal models and longitudinal datasets, in order to characterize its robustness and predictive competence. In this sense, it would be of relevance to validate the identified epicenter regions, and to evaluate the ability of the model to predict future MP deposition states, and/or validate the estimated production and clearance rates. Additionally, structural connectivity information should be continuously improved, along with available anatomical network reconstruction techniques. The influence on the model variables of other genes identified as essential Aß modulators (e.g. BCHE, MGAT3 and CD33 genes [35]–[37], [54]) should be also explored. Since the formulated model (*Equation 1*) presents the same mathematical structure as the well described predator-prey Lotka-Volterra systems [86], [87], it would be interesting to analyze the individual anatomical stability conditions supporting the intra-brain propagation of the MP factors. Finally, and based on the fact that MP are not the unique factors associated to neurodegenerative progression, more advanced models should be directed towards characterizing MP effects in conjunction with other pathological mechanisms, such as metabolic, vascular and functional dysregulations.

## Methods

### Ethics statement

The study was conducted according to Good Clinical Practice guidelines, the Declaration of Helsinki, US 21CFR Part 50 – Protection of Human Subjects, and Part 56 – Institutional Review Boards, and pursuant to state and federal HIPAA regulations [88]. Study subjects and/or authorized representatives gave written informed consent at the time of enrollment for sample collection and completed questionnaires approved by each participating sites Institutional Review Board (IRB) [88]. The authors obtained approval from the ADNI Data Sharing and Publications Committee for data use [89] and publication [90].

### Intra-brain Epidemic spreading model (ESM) of misfolded proteins (MP) propagation/deposition

Here we consider the brain as a system with *N* structurally interconnected gray matter regions, where each region *i* (*i = 1..N*) is characterized by its temporal probability (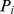) of MP burden. The dynamic behaviour of this system, in terms of MP propagation and deposition, will depend on the interactions between the MP “infested” and “non-infested” regions, where temporal changes in the regional 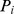 values can be described by the non-linear differential model:
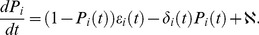
(1)


The first term on the right side of Equation (1) represents the regional probability of receiving MP infectious-like agents (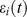) if region *i* is “non-infested” (which happens with probability 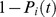). The second term, corresponds to the probability of being clean of MP at time t (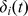) if region *i* was “infested” before t (which happens with probability 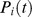). The last term, represents an additive noise (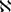) due to possible stochastic processes, such as natural stochastic factors mediating MP aggregation mechanisms [91] or unknown effects of therapeutic medications.

In the traditional epidemic disease spreading framework, self infection processes are not considered (i.e. the 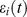 term only quantifies the possibility of receiving infectious-like agents from other system entities/nodes). However, in our case, each system entity corresponds to a macroscopic brain region, which comprises several neuronal groups (a direct consequence of the limited PET/MRI spatial resolution and available gray matter parcellation schemes, e.g., [42], [92]). Then, we need to consider the fact that a particular “infested” sub-region in *i* can potentially “infect” neighboring sub-regions. Therefore, 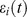 is modeled as the probabilistic accumulation of exogenous and endogenous infectious-like factors:
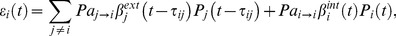
(2)where 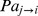 is the weighted anatomical connection probability between the regions *j* and *i* (see *Anatomical connection probability* subsection), 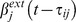 is the extrinsic “infection” rate of region *j* at time 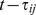, 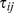 is the delay corresponding to the time the soluble MP takes to depart from *j*, with propagation velocity *V_MP_* [20], and cover the connection distance 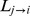; 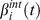 is the intrinsic “infection” rate of region *i* at *t*.

The distinction between extrinsic and intrinsic regional “infection” rates reflects the fact that the total soluble MP produced at a given region is subdivided in two competing processes: the diffusion towards the region's external space (contributing to the global MP expansion) and the molecules staying inside the region (contributing to the production of new seeds and participating in local aggregation mechanisms). As mentioned, soluble MP diffuse from regions of higher concentration to regions of lower concentration. Thus, a high inequality in the deposition levels of all the considered gray matter regions will cause an increase in the extrinsic propagation of soluble MP across the entire brain, and a decrease in the intrinsic fraction of soluble MP that stays in each seed region. These effects are characterized by the relations:
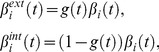
(3)where 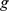 is a global tuning variable that quantifies the temporal MP deposition inequality among the different brain regions, and 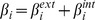 is the total “infection” rate of the region *i*. We assume 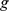 as the *Gini* coefficient [93], a well established measure of statistical dispersion in a given system, with value 0 reflecting perfect equality and value 1 corresponding to a complete inequality. 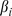 is defined as a sigmoid function of the regional MP deposition probability, with a high MP deposition probability implying a high probability of producing new infectious-like factors (according to the seeding/nucleation mechanisms, see [94], [95]):
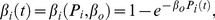
(4)being 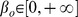 an unknown constant parameter.

Similarly to 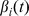, the regional probability of being clean of MP after “infection” (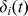) is expressed as a function of 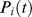 and a constant parameter. However, because MP deposition implies inflammation processes and cellular death, the regional capacity to clear/remove infectious-like agents will decrease with the increase in MP deposition, following a decreasing exponential relationship:
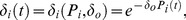
(5)where 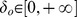 is also an unknown constant parameter. We hypothesize that 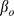 and 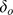 will depend on the specific MP under study, as well as on the individual characteristics (e.g. genetic properties, life style, environmental conditions).

The additive noise (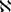) in Equation 1 is assumed to follow a Gaussian distribution with unknown mean μ and standard deviation σ. From equations (1)–(5) we see that the proposed model depends on four unknown parameters (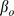, 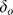, μ and σ), which will control the continuous competition between the MP infectious-like agents, the system's clearance response, and the unidentified random processes or external inputs.

### Data description and processing

#### Study participants

Dataset 1: This study used 733 individual data from the Alzheimer's Disease Neuroimaging Initiative (ADNI) [88]. The ADNI was launched in 2003 by the National Institute on Aging (NIA), the National Institute of Biomedical Imaging and Bioengineering (NIBIB), the Food and Drug Administration (FDA), private pharmaceutical companies and non-profit organizations, as a $60 million, 5-year public-private partnership. The primary goal of ADNI has been to test whether serial magnetic resonance imaging (MRI), positron emission tomography (PET), other biological markers, and clinical and neuropsychological assessment can be combined to measure the progression of mild cognitive impairment (MCI) and early Alzheimer's disease (AD). Determination of sensitive and specific markers of very early AD progression is intended to aid researchers and clinicians to develop new treatments and monitor their effectiveness, as well as lessen the time and cost of clinical trials. The Principal Investigator of this initiative is Michael W. Weiner, MD, VA Medical Center and University of California – San Francisco. ADNI is the result of efforts of many co-investigators from a broad range of academic institutions and private corporations, and subjects have been recruited from over 50 sites across the U.S. and Canada. The initial goal of ADNI was to recruit 800 subjects but ADNI has been followed by ADNI-GO and ADNI-2. To date these three protocols have recruited over 1500 adults, ages 55 to 90, to participate in the research, consisting of cognitively normal older individuals, people with early or late MCI, and people with early AD. The follow up duration of each group is specified in the protocols for ADNI-1, ADNI-2 and ADNI-GO. Subjects originally recruited for ADNI-1 and ADNI-GO had the option to be followed in ADNI-2 [88]. Written informed consent was obtained from all participants before protocol-specific procedures were performed [88]. For up-to-date information, see www.adni-info.org.

See Table S1 for demographic and clinical characteristics of the included ADNI subjects.

Dataset 2: In addition, this study used the data of 60 young healthy subjects, from the CMU-60 DSI Template (http://www.psy.cmu.edu/~coaxlab/?page_id=423). The CMU-60 DSI Template is a freely available map of reconstructed fiber orientations from very high angular resolution diffusion MRI data, acquired with a 257-direction diffusion spectrum imaging (DSI) sequence. It was developed by The Cognitive Axon (CoAx) Lab, in the Center for the Neural Basis of Cognition and Department of Psychology at Carnegie Mellon University. The 60 subjects (29 male and 31 female) were recruited from the local Pittsburgh community and the Army Research Laboratory in Aberdeen Maryland. All subjects were neurologically healthy, with no history of either head trauma or neurological or psychiatric illness. Subject ages ranged from 18 to 45 years of age (mean 26 ± 6) at the time of scanning. All acquisition and preprocessing steps described below were performed originally by the members of the CoAx Lab.

#### Image acquisition

Dataset 1: A 370 MBq (10 mCi+/-10%) bolus injection of AV-45 was administered to each participant, and 20 minute continuous brain PET imaging were acquired approximately 50 minutes post-injection. The images were reconstructed immediately after the 20 minute scan, and when motion artifact was detected, another 20 minute continuous scan was acquired.

Dataset 2: Participants were scanned on a Siemen's Verio 3T system in the Scientific Imaging & Brain Research (SIBR) Center at Carnegie Mellon University using a 32-channel head coil. 50 min, 257-direction DSI scan using a twice-refocused spin-echo EPI sequence and multiple q values (TR  = 9,916 ms, TE  = 157 ms, voxel size  = 2.4×2.4×2.4 mm, FoV  = 231×231 mm, b-max  = 5,000 s/mm^2^, 51 slices) were collected. Head-movement was minimized during the image acquisition through padding supports and all subjects were confirmed to have minimal head movement during the scan prior to inclusion in the template.

#### Image processing

Dataset 1: For each individual PET acquisition, images were initially preprocessed according to four main steps [96]: 1) dynamic co-registration (separate frames were co-registered to one another lessening the effects of patient motion), 2) across time averaging, 3) re-sampling and reorientation from native space to a standard voxel image grid space (“AC-PC” space), and 4) spatial filtering to produce images of a uniform isotropic resolution of 8 mm FWHM. Next, all images were spatially normalized to the MNI space [97].

Dataset 2: All images were processed using a q-space diffeomorphic reconstruction method described previously [98]. This method uses a non-linear coregistration approach (ICBM-152 space template regularization, 16 non-linear iterations) that registers the voxel-coordinate into MNI space while also maintaining distortion of the q-space vector during the normalization process. From here, orientation distribution functions (ODFs) were reconstructed to spatial resolution of 2 mm^3^. The final template image was created by averaging the ODF maps across all 60 subjects, constituting a detailed and unbiased representative map of the nervous fiber orientations in the young healthy brain.

#### Regional Aß deposition patterns

Considering the Cerebellum as an Aß non-specific binding reference, individual Aß deposition probabilities for 78 regions covering all the brain's gray matter [42] were calculated. First, a bootstrap sampling, consisting of 40,000 randomized sub-samples of the Cerebellum's PET signal values, was used to approximate the extreme value distribution for the maximum values at this region (denoted as 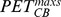). Next, the maximum likelihood parameters associated to the empirical 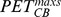 distribution were used to calculate the cumulative distribution value of each brain voxel *r* with signal intensity 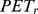. Note that this value will be expressing the probability of the presence, at voxel *r,* of an equal or higher value than all the possible maximum values within the Cerebellum, i.e. 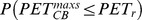. Then, the Aß deposition probability for a given region *i* (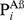) was calculated as:
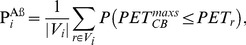
(6)where 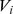 is the set of voxels demarked by the region *i*. Finally, these 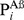 values (i = 1..78) were assumed to represent the individual Aß deposition patterns.

#### Anatomical connection probability

Probabilistic axonal connectivity values between each brain voxel and the surface of each considered gray matter region (voxel-region connectivity) were estimated using a fully automated fiber tractography algorithm [83] and the intravoxel fiber ODFs of the CMU-60 DSI Template. A maximum of 500 mm trace length and a curvature threshold of ±90 were imposed as tracking parameters.

Based on the resulting voxel-region connectivity maps, the anatomical connection probability between any pair of regions *i* and *j* (*ACP_ij_ ≡ ACP_ji_*) was calculated as the maximum voxel-region connectivity value between both regions. The *ACP* measure [83] reflects the degree of evidence supporting the existence of each hypothetical white matter connection, independently of the density/strength of this connection, and is thus a measure of low susceptibility to gross fiber degeneration effects related to aging processes. Self connections were considered with *ACP_ii_*  = 1.

Effective anatomical distances to the outbreak regions were estimated as the length of the shortest path (in terms of ACP values) linking region *i* with the posterior and anterior cingulate cortices [49].

#### CSF measures

In addition to the neuroimaging data, CSF Aß^1-42^, t-tau and p-tau^181^ measurements were acquired for 307 subjects belonging to *Dataset 1*. This subsample comprised HC (n = 78), EMCI (n = 150), LMCI (n = 58) and AD (n = 21) subjects. The xMAP Luminex platform and Innogenetics/Fujirebio AlzBio3 immunoassay kits were used following the SOP in place at the UPenn/ADNI Biomarker Laboratory [68], [99], [100]. Further details on data collection can be found at http://www.adni-info.org. Data were preprocessed as described in [68], [100].

#### Model exploration/validation with simulated data

Finally, we tried to reproduce the individual PET-based Aß deposition patterns from remote “non-infectious” states. All brain regions, or their combinations up to a maximum of 6 regions (i.e. a total of 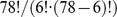 = 256851595 combinations), were considered as possible candidates to start the Aß propagation. For each set of sAß spreading seed regions, multiple lifetime trajectories of Aß propagation were simulated (Equation 1). Each simulated trajectory consisted of 50 continuous years of hypothetical Aß deposition patterns, with each pattern corresponding to a 1 day period. Similar to the creation of the Aß reference patterns, each simulated Aß time point pattern (representing one day across the 50 years) consisted of 78 regional probability values, reflecting the local Aß “infection” levels. For each subject *i* (*i* = [1,733]), we explored iteratively across the parameters space (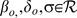, assuming positive values) and the corresponding 50 years trajectories until we identified the set 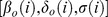 that minimized, at a unique one day time point t(i), the Euclidean distance between the reference and the simulated Aß deposition patterns. Note that, ideally, this time point t(i) should match the ^18^F-AV-45 PET scan acquisition day for subject *i* (Figure 1).

Once we selected the most likely “infectious” seed regions that best explained the reference Aß deposition patterns across the study cohort, as well as the optimized individual parameters 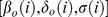 and corresponding t(i), we marginalized across all possible regional *P* values to obtain the individualized global Aß production and clearance rates: 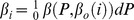 and 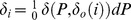, respectively. The individual onset ages of Aß binding were also calculated as the difference between the chronological ages and the optimum time points t(i). In sum, for each subject, the individual Aß propagation/deposition history was characterized by the set of model parameters 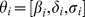 and the associated onset age (Figure 1).

### Statistical analysis

#### Model cross-validation analysis (Results, first subsection, Figure 2C)

Repeated random sub-sampling was used to split each clinical group dataset into training and test data (both data with same sample sizes, n/2). For each split, the model was fitted to the training data, and predictive accuracy (explained variance) was assessed using the test data.

#### Model variables impact on clinical states (Results, third subsection, Figure 4A)

The LMG metric [101] was used, in combination with a Multinomial Logistic Regression, to assess relative importance of regressors. LMG quantify the proportionate contribution of each regressor to the global coefficient of determination (see Grömping, 2006, for review on relative importance metrics). Bootstrapping was used to construct the sampling distributions of the LMG measures and the associated empirical confidence intervals [102].

#### APOE E4, demographic variables and model-based variables (Results, fourth subsection, Figure 5)

Seven-way ANOVA was used to assess predictors association with each model variable. Pair-wise predictor interactions were considered.

*Model-based and demographic variables association with CSF measures* (*Results*, final subsection, Figure 6): Seven-way ANOVA was used to assess predictors association with each CSF measure.

One-tailed Student's test was used for all the between group comparisons in the study.

## Supporting Information

Text S1 Analyzing the ratio between global Aß production and clearance rates.
(DOCX)

Text S2 Complete list of ADNI investigators.
(PDF)

Figure S1 Relations between effective anatomical distances to all brain regions and Aß deposition levels. A) Correlations values conserving the original order of the regions in the atlas. B) Absolute correlations after sort the regions from maximum to minimum values, in order to illustrate their natural order as potential propagation seeds. In A) and B), abbreviations are: ACC as anterior cinculate cortex, and PCC as posterior cingulated cortex.
(TIF)

Figure S2 Regional Aß deposition probability for the different clinical groups vs effective anatomical distance to the outbreak regions.
(TIF)

Figure S3 Regional Aß arriving times vs anatomical connectivity degrees, for different Aß probability thresholds (i.e. 0.1, 0.5 and 0.9). Seed regions were not included.
(TIF)

Figure S4 Mean (± standard error) ratio between Aß production and clearance rates for the different clinical groups (adjusted for gender and educational level). *p<0.05, **p<10^−4^, One-tailed Student's *t*-test.
(TIF)

Table S1 Demographic and clinical characteristics of included ADNI subjects.
(DOCX)

Table S2 Different models explaining Aß deposition or cortical atrophy associated to AD.
(DOCX)

Table S3 Examples of considered seed regions for starting Aß propagation.
(DOCX)

Table S4 Contribution of Human Anatomical Connectivity information on MP modeling.
(DOCX)

Table S5 Prediction accuracy values obtained with the ESM via a repeated random sub-sampling cross-validation.
(DOCX)

Table S6 Contributions of Effective Anatomical Distance and Spatial Proximity to identified Epicenters on regional Aß levels.
(DOCX)

Table S7 Clinical diagnosis explained by model variables (LMG metric results, after adjusting for gender and educational level).
(DOCX)

Table S8 Model variables differences between clinical groups (*t*-test results, after adjusting for gender and educational level).
(DOCX)

Table S9 Model variables explained by APOE e4 genotype, gender and educational level (ANOVA results).
(DOCX)

Table S10 Model variables differences between APOE e4 genotype groups (*t*-test results, after adjusting for gender and educational level).
(DOCX)

Table S11 Model variables differences between genders (*t*-test results, after adjusting for APOE e4 genotype and educational level).
(DOCX)

Table S12 CSF measures explained by model variables, gender, age and educational level (ANOVA results).
(DOCX)
