# Supplementary material for: Epidemic Spreading Model to Characterize Misfolded Proteins Propagation in Aging and Associated Neurodegenerative Disorders
Source: PLoS Comput Biol. 2014 Nov 20;10(11):e1003956. doi: 10.1371/journal.pcbi.1003956 (PMC4238950; doi:10.1371/journal.pcbi.1003956)
Supplement: Text S1 — Analyzing the ratio between global Aß production and clearance rates. (DOCX) [file pcbi.1003956.s001.docx]

**Text S1**.

We attribute the observed decrease in lifetime Aß production, from healthy to pathological states, to a “saturation” effect: the early onset of Aß propagation and the strong deficiency in Aß clearance elicit an early abnormal Aß deposition pattern and subsequently a decrease in Aß agents recirculation and production. The latter effect might be causally involved in the significant decrease of cerebrospinal fluid (CSF) Aß concentrations observed for MCI-AD patients (see also *Modulatory impact of Aß propagation/deposition history on CSF Aß^1-42^, t-tau and p-tau levels* subsection, *Results* section). To test this “saturation” hypothesis, we calculated the ratio between the individualized global Aß production and clearance rates. We observed that non-healthy subjects present a significantly higher ratio between Aß production and clearance rates (Figure S4). This suggests that even when lifetime Aß production rate decreases for the non-healthy states, such effect might be clinically irrelevant compared with the associated more pronounced lifetime decreases in Aß clearance, and the newly produced Aß agents continue to induce higher deposition levels. Together, these results indicate that a malfunctioning on the Aß clearance system of the future non-healthy subjects greatly influences the dynamic interactions between the infectious-like agents and the brain’s clearance responses, leading to abnormal Aß deposition patterns.
