# Supplementary figures and images for: Epidemic Spreading Model to Characterize Misfolded Proteins Propagation in Aging and Associated Neurodegenerative Disorders
Source: PLoS Comput Biol. 2014 Nov 20;10(11):e1003956. doi: 10.1371/journal.pcbi.1003956 (PMC4238950; doi:10.1371/journal.pcbi.1003956)

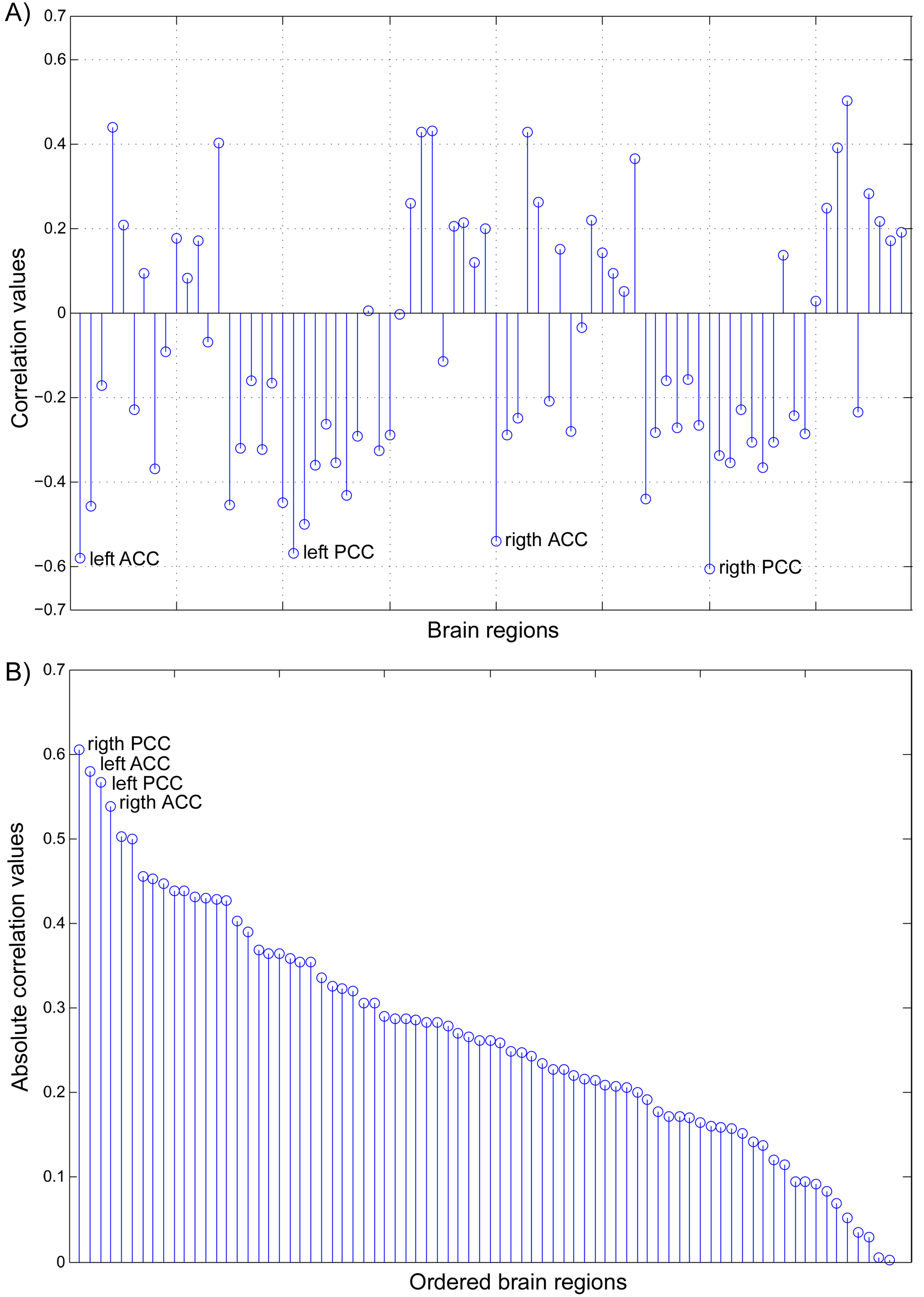

Supplement: Figure S1 — Relations between effective anatomical distances to all brain regions and Aß deposition levels. A) Correlations values conserving the original order of the regions in the atlas. B) Absolute correlations after sort the regions from maximum to minimum values, in order to illustrate their natural order as potential propagation seeds. In A) and B), abbreviations are: ACC as anterior cinculate cortex, and PCC as posterior cingulated cortex. (TIF) [file pcbi.1003956.s003.tif]

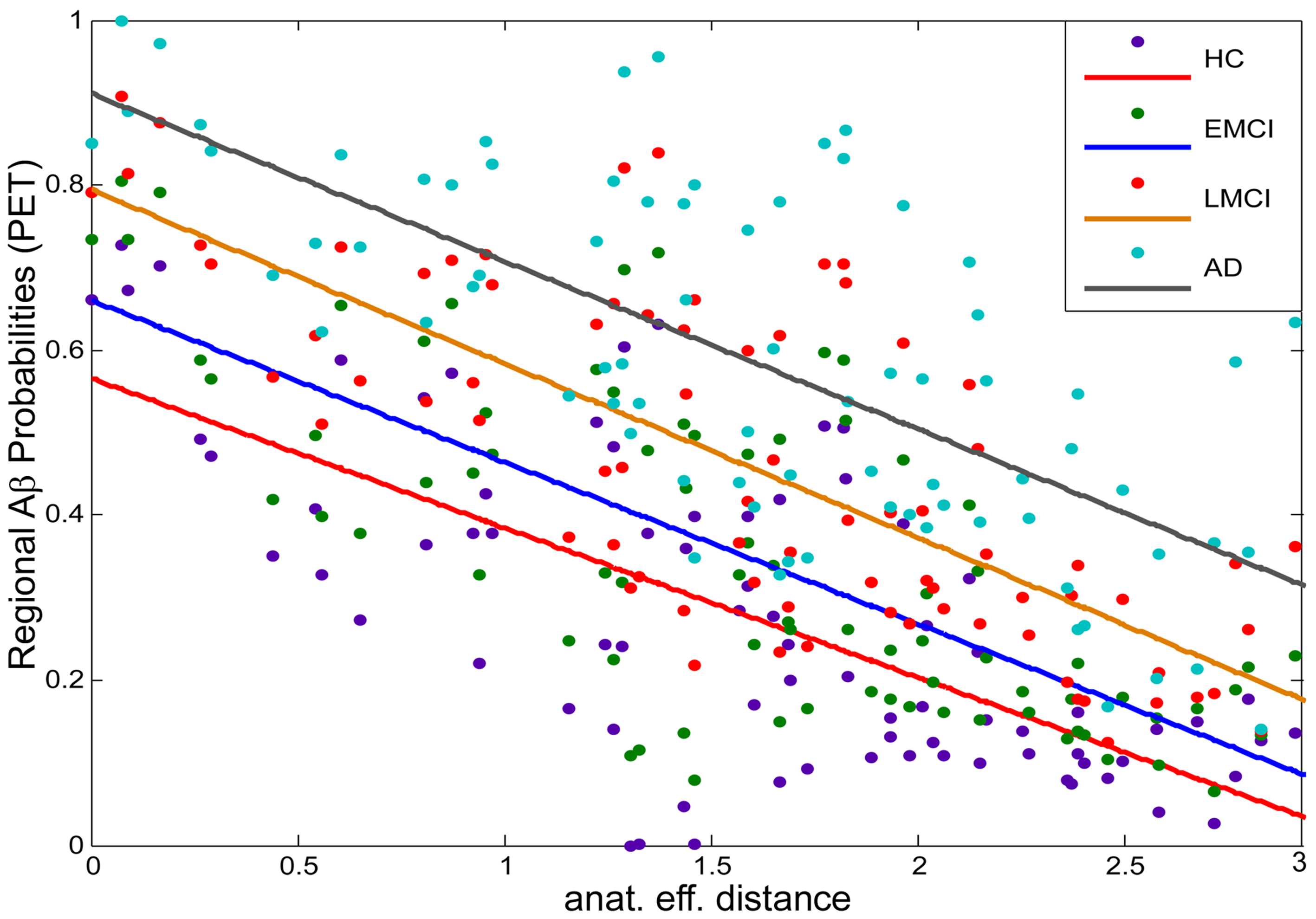

Supplement: Figure S2 — Regional Aß deposition probability for the different clinical groups vs effective anatomical distance to the outbreak regions. (TIF) [file pcbi.1003956.s004.tif]

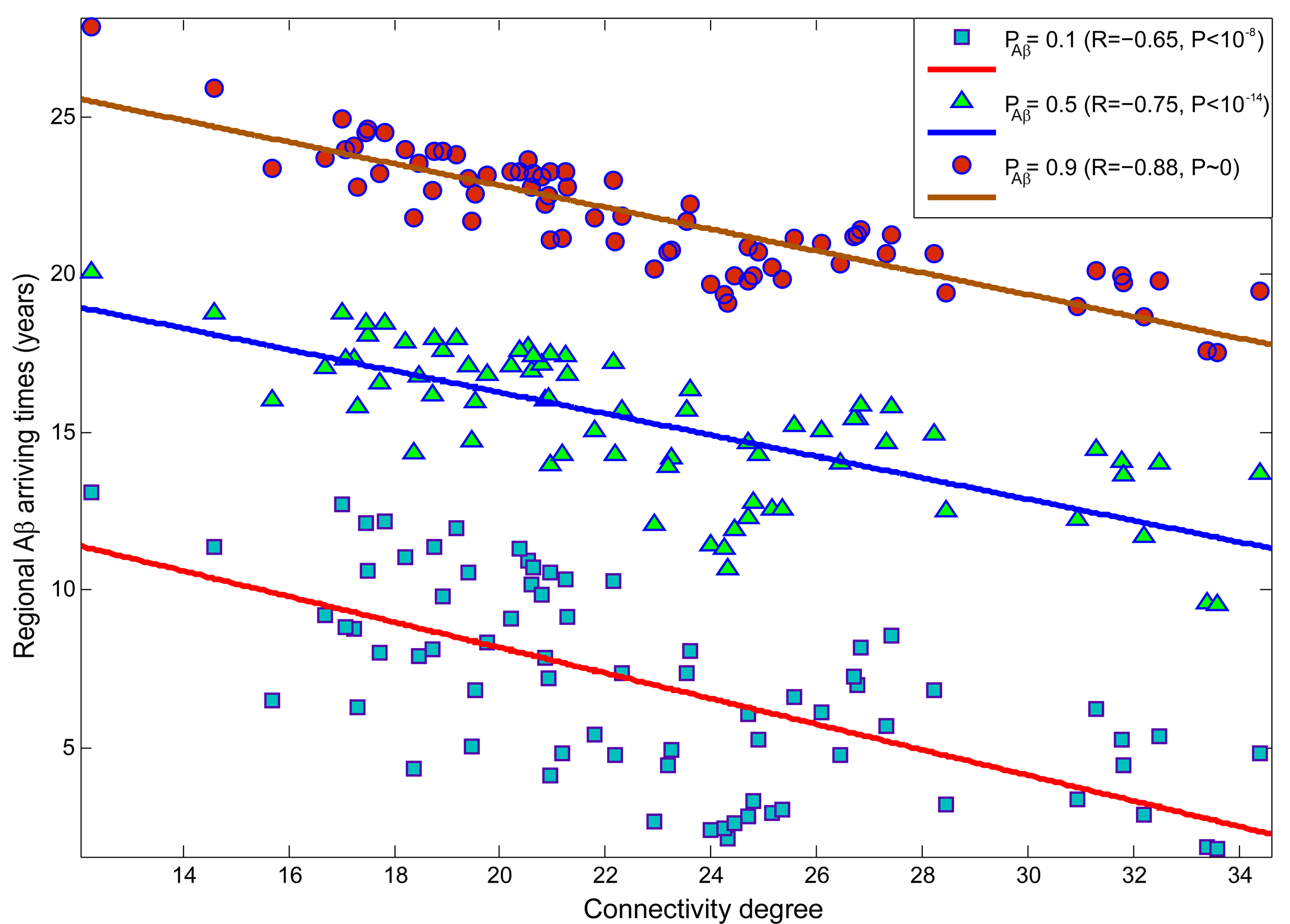

Supplement: Figure S3 — Regional Aß arriving times vs anatomical connectivity degrees, for different Aß probability thresholds (i.e. 0.1, 0.5 and 0.9). Seed regions were not included. (TIF) [file pcbi.1003956.s005.tif]

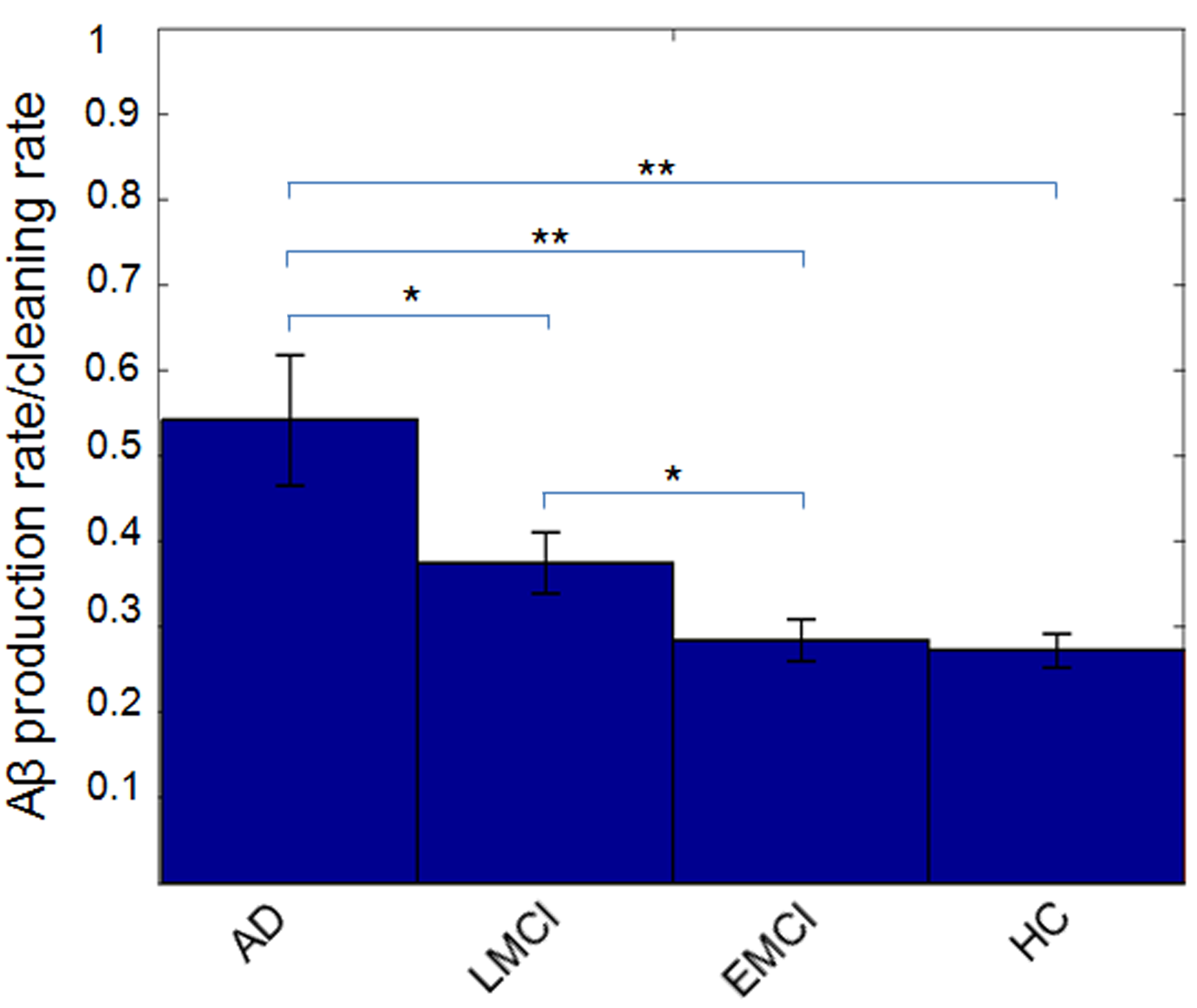

Supplement: Figure S4 — Mean (± standard error) ratio between Aß production and clearance rates for the different clinical groups (adjusted for gender and educational level). *p<0.05, **p<10−4, One-tailed Student's t-test. (TIF) [file pcbi.1003956.s006.tif]
