# Supplementary material for: Epidemic Spreading Model to Characterize Misfolded Proteins Propagation in Aging and Associated Neurodegenerative Disorders
Source: PLoS Comput Biol. 2014 Nov 20;10(11):e1003956. doi: 10.1371/journal.pcbi.1003956 (PMC4238950; doi:10.1371/journal.pcbi.1003956)
Supplement: Table S1 — Demographic and clinical characteristics of included ADNI subjects. (DOCX) [file pcbi.1003956.s007.docx]

**Table S1**.

| **Characteristics** | **AD**  (n = 111) | **LMCI**  (n = 196) | **EMCI**  (n = 233) | **HC**  (n = 193) |
| --- | --- | --- | --- | --- |
| Women | 47(42 %) | 88 (44 %) | 100(43 %) | 94 (49 %) |
| Age (years) | 74.30 (7.92) | 71.72 (7.71) | 70.77 (7.18) | 73.88(5.73) |
| APOE e4 (1 copy) | 61 (55 %) | 108 (55 %) | 94 (40 %) | 50 (26 %) |
| APOE e4 (2 copies) | 19 (17 %) | 30 (15 %) | 14 (6 %) | 4 (2 %) |
| Education (years) | 15.82 (2.63) | 16.06(2.81) | 16.00(2.65) | 16.46(2.71) |
| MMSE^1^ | 22.82(2.01) | 27.60(1.80) | 28.39(1.52) | 29.10(1.14) |
| CDR^2^ | 4.45(1.66) | 1.68(0.99) | 1.26(0.76) | 0.02(0.11) |

^1^MMSE, mini-mental state examination; ^2^CDR, clinical dementia rating. Data are number (%) or mean (std).
