# Supplementary material for: Epidemic Spreading Model to Characterize Misfolded Proteins Propagation in Aging and Associated Neurodegenerative Disorders
Source: PLoS Comput Biol. 2014 Nov 20;10(11):e1003956. doi: 10.1371/journal.pcbi.1003956 (PMC4238950; doi:10.1371/journal.pcbi.1003956)
Supplement: Table S2 — Different models explaining Aß deposition or cortical atrophy associated to AD. (DOCX) [file pcbi.1003956.s008.docx]

**Table S2**.

| **Model** | **Variable** | **Explained variance** (%) | **Sample size** | **# of regions** |
| --- | --- | --- | --- | --- |
| APOE [1] | Total Aß deposition | 10.7 | 555 | 1 (average of 5 regions) |
| BCHE [1] | Total Aß deposition | 4.3 | 555 | 1 (average of 5 regions) |
| Intrinsic functional connectivity [2] | Regional Cortical atrophy | 39 | 24 | 1128 |
| Network diffusion [3] | Regional Cortical atrophy | 5.29 | 18 | 90 |
| Network diffusion [3] (evaluated in this study)^1^ | Regional Aß deposition | 51.41 (HC),  53.87 (EMCI),  56.74 (LMCI),  46.37 (AD) | 193 (HC),  233 (EMCI),  196 (LMCI),  111 (AD) | 78 |
| Epidemic spreading (this study) | Regional Aß deposition | 27.48 (HC),  31.80 (EMCI),  32.32 (LMCI),  31.56 (AD) | 193 (HC),  233 (EMCI),  196 (LMCI),  111 (AD) | 78 |

* Explained variances (%) were calculated as the square of the correlation between estimated and reference values, multiplied by 100.

^1^The Network Diffusion Model (NDM) [3] was applied with a purpose of inter-methods comparison. We used the same set of subjects (Dataset 1, n=733) and connectivity matrix obtained for the CMU-60 DSI template (Dataset 2). Importantly, as this model was designed to describe the temporal regional MPs concentrations (instead of the regional probabilities), we used regional Standardized Uptake values (SUVR) to create the reference/target Aß deposition patterns (values were normalized between zero and one). In line with our method, this model also identified the posterior and anterior cingulate cortices as the most probable starting seed regions for the Aß propagation process. However, even when the obtained mean regional explained variance for the NDM was around 27-33 %, the associated *root mean square errors* (RMSEs) were considerable high, reflecting high absolute differences between estimated and reference Aß concentration patterns. For instance, compared with ESM, the NDM obtained significantly higher RMSEs values (P=3.60x10^-207^, Z=-30.69; based on Wilconson rank sum test). In addition, Akaike Information Criterion (AIC) values evaluated for both models revealed a significantly lower accuracy performance for the NDM, independently of the number of models parameters (P=7.13x10^-8^, Z=-5.26; based on Wilconson rank sum test). We noted that although the NDM is capable of dispersing the initial infectious-like factors from the seed regions to the rest of the brain network, such dispersion is at the expense of the local concentrations, which after the initial exchange decreases continuously. As a consequence, the total Aß concentration is never higher than the “injected” amount. After a given time, the model cannot keep the factors propagation. In general, these limitations might be reflecting the absence of a source term in the NDM.

References:

[1] V. Ramanan, S. Risacher, K. Nho, S. Kim, S. Swaminathan, L. Shen, T. Foroud, H. Hakonarson, M. Huentelman, P. Aisen, R. Petersen, R. Green, C. Jack, R. Koeppe, W. Jagust, M. Weiner, and A. Saykin, “APOE and BCHE as modulators of cerebral amyloid deposition: a florbetapir PET genome-wide association study,” *Mol. Psychiatry*, 2013.

[2] J. Zhou, E. D. Gennatas, J. H. Kramer, B. L. Miller, and W. W. Seeley, “Predicting Regional Neurodegeneration from the Healthy Brain Functional Connectome,” *Neuron*, vol. 73, no. 6, pp. 1216–1227, Mar. 2012.

[3] A. Raj, A. Kuceyeski, and M. Weiner, “A Network Diffusion Model of Disease Progression in Dementia,” *Neuron*, vol. 73, no. 6, pp. 1204–1215, Mar. 2012.
