# Supplementary material for: Epidemic Spreading Model to Characterize Misfolded Proteins Propagation in Aging and Associated Neurodegenerative Disorders
Source: PLoS Comput Biol. 2014 Nov 20;10(11):e1003956. doi: 10.1371/journal.pcbi.1003956 (PMC4238950; doi:10.1371/journal.pcbi.1003956)
Supplement: Table S3 — Examples of considered seed regions for starting Aß propagation. (DOCX) [file pcbi.1003956.s009.docx]

**Table S3**.

| **Infectious seeds** (reference source) | **Explained variance** (%) |
| --- | --- |
| Posterior and anterior cingulate cortices (automatic selection, this study) | 35.8±1.22 |
| Posterior cingulate cortex and temporal-parietal junction [1] | 17.5±1.87 |
| Angular gyrus [2] | 14.7±0.45 |
| Amygdala, orbitofrontal cortex and hippocampus [3] | 4.33±0.37 |
| Entorhinal cortex [4] | 3.47±0.88 |

*Data are regional deposition explained variance ± 0.975 quantile.

References:

[1] N. Villain, G. Chételat, B. Grassiot, P. Bourgeat, G. Jones, K. a Ellis, D. Ames, R. N. Martins, F. Eustache, O. Salvado, C. L. Masters, C. C. Rowe, and V. L. Villemagne, “Regional dynamics of amyloid-β deposition in healthy elderly, mild cognitive impairment and Alzheimer’s disease: a voxelwise PiB-PET longitudinal study.,” *Brain*, vol. 135, no. Pt 7, pp. 2126–39, Jul. 2012.

[2] J. Zhou, E. D. Gennatas, J. H. Kramer, B. L. Miller, and W. W. Seeley, “Predicting Regional Neurodegeneration from the Healthy Brain Functional Connectome,” *Neuron*, vol. 73, no. 6, pp. 1216–1227, Mar. 2012.

[3] J. Sepulcre, M. R. Sabuncu, A. Becker, R. Sperling, and K. a Johnson, “In vivo characterization of the early states of the amyloid-beta network.,” *Brain*, vol. 136, no. Pt 7, pp. 2239–52, Jul. 2013.

[4] B. E. Braak H, “Neuropathological stageing of Alzheimer-related changes,” *Acta Neuropathol.*, vol. 82, no. 4, pp. 239–59, 1991.
