# Supplementary material for: Epidemic Spreading Model to Characterize Misfolded Proteins Propagation in Aging and Associated Neurodegenerative Disorders
Source: PLoS Comput Biol. 2014 Nov 20;10(11):e1003956. doi: 10.1371/journal.pcbi.1003956 (PMC4238950; doi:10.1371/journal.pcbi.1003956)
Supplement: Table S4 — Contribution of Human Anatomical Connectivity information on MP modeling. (DOCX) [file pcbi.1003956.s010.docx]

**Table S4.**

| **Group** | ESM performance | Aß level vs effective ant. distance to Epicenter |
| --- | --- | --- |
| HC | 4.72 (1.16 x 10^-6^) | 5.42 (1.32 x 10^-12^) |
| EMCI | 8.21 (1.05 x 10^-16^) | 5.33 (2.66 x 10^-15^) |
| LMCI | 11.84 (1.08 x 10^-32^) | 4.82 (4.90 x 10^-14^) |
| AD | 11.71 (5.54 x 10^-32^) | 4.48 (1.26 x 10^-12^) |

Data are *Z* value (significance value) calculated by comparing, via a *Z* test, the predictive correlation obtained using the human anatomical connectivity matrix vs the distribution of predictive correlation values obtained using non-informative connectivity matrices. A positive *Z* value indicates a higher absolute correlation value obtained with the human anatomical connectivity information. Each non-informative matrix (100 in total) corresponded to a different randomization of the original connectivity matrix, preserving its weight, degree and strength distributions (to create these we used the *Brain Connectivity Toolbox*, available at https://sites.google.com/site/bctnet/, function *null_model_und_sign*).
