# Supplementary material for: Epidemic Spreading Model to Characterize Misfolded Proteins Propagation in Aging and Associated Neurodegenerative Disorders
Source: PLoS Comput Biol. 2014 Nov 20;10(11):e1003956. doi: 10.1371/journal.pcbi.1003956 (PMC4238950; doi:10.1371/journal.pcbi.1003956)
Supplement: Table S5 — Prediction accuracy values obtained with the ESM via a repeated random sub-sampling cross-validation. (DOCX) [file pcbi.1003956.s011.docx]

**Table S5**.

| **Group** | **Accuracy** (%) |
| --- | --- |
| HC | 40.7 (36.3, 45.0) |
| EMCI | 45.9 (40.6, 51.8) |
| LMCI | 51.6 (46.5, 57,7) |
| AD | 31.4 (28.4, 34.2) |

Data are prediction accuracy (95 % confidence interval).
