# Supplementary material for: Epidemic Spreading Model to Characterize Misfolded Proteins Propagation in Aging and Associated Neurodegenerative Disorders
Source: PLoS Comput Biol. 2014 Nov 20;10(11):e1003956. doi: 10.1371/journal.pcbi.1003956 (PMC4238950; doi:10.1371/journal.pcbi.1003956)
Supplement: Table S6 — Contributions of Effective Anatomical Distance and Spatial Proximity to identified Epicenters on regional Aß levels. (DOCX) [file pcbi.1003956.s012.docx]

**Table S6.**

| **Group** | Effective Anatomic Distance To Epicenter | Spatial distance to Epicenter (i.e. Euclidean distance) | Effective Anatomic Distance To Epicenter (controlling first by Spatial Proximity) |
| --- | --- | --- | --- |
| HC | 48.67 (1.26 x 10^-12^) | 29.85 (2.28 x 10^-7^) | 27.91 (7.88 x 10^-7^) |
| EMCI | 52.83 (4.90 x 10^-14^) | 29.68 (2.51 x 10^-7^) | 33.38 (3.75 x 10^-8^) |
| LMCI | 56.27 (2.66 x 10^-15^) | 24.59 (3.88 x 10^-6^) | 42.16 (1.68 x 10^-10^) |
| AD | 48.61 (1.32 x 10^-12^) | 15.96 (2.89 x 10^-4^) | 40.46 (5.07 x 10^-10^) |

Data are prediction accuracy values (significance value). See *Predicting regional Aß arrival time with effective anatomical distance to outbreak region* subsection, in *Results* section.
