# Supplementary material for: Epidemic Spreading Model to Characterize Misfolded Proteins Propagation in Aging and Associated Neurodegenerative Disorders
Source: PLoS Comput Biol. 2014 Nov 20;10(11):e1003956. doi: 10.1371/journal.pcbi.1003956 (PMC4238950; doi:10.1371/journal.pcbi.1003956)
Supplement: Table S7 — Clinical diagnosis explained by model variables (LMG metric results, after adjusting for gender and educational level). (DOCX) [file pcbi.1003956.s013.docx]

**Table S7**.

| **Modulators** | **Aß Production rate** ($\beta$) | **Aß Clearance rate**  ($\delta$) | **Noise**  (σ) | **Onset Age**  (Age_onset_) |
| --- | --- | --- | --- | --- |
| Clinical diagnosis (HC, EMCI, LMCI, AD) | 2.15(-0.75,3.77) | 8.45(4.88,11.89) | 0(-3.73,0) | 6.77(3.41,9.88) |

Data are explained variance (95 % confidence interval).
