# Supplementary material for: Epidemic Spreading Model to Characterize Misfolded Proteins Propagation in Aging and Associated Neurodegenerative Disorders
Source: PLoS Comput Biol. 2014 Nov 20;10(11):e1003956. doi: 10.1371/journal.pcbi.1003956 (PMC4238950; doi:10.1371/journal.pcbi.1003956)
Supplement: Table S8 — Model variables differences between clinical groups (t-test results, after adjusting for gender and educational level). (DOCX) [file pcbi.1003956.s014.docx]

**Table S8**.

| **Compared groups** | **Aß Production rate**  ($\beta$) | **Aß Clearance rate**  ($\delta$) | **Noise**  (σ) | **Onset Age**  (Age_onset_) |
| --- | --- | --- | --- | --- |
| HC-EMCI | 1.75(0.04) | 2.47(0.006) | -0.21(0.41) | 5.57(2.19 x10^-8^) |
| EMCI-LMCI | 2.35(0.009) | 4.58(3.04x10^-6^) | -0.17 (0.43) | 2.30(0.01) |
| LMCI-AD | 1.01(0.16) | 1.97(0.02) | -0.50(0.30) | 0.51(0.30) |

Data are test statistic (statistical significance, i.e., P values).
