# Supplementary material for: Epidemic Spreading Model to Characterize Misfolded Proteins Propagation in Aging and Associated Neurodegenerative Disorders
Source: PLoS Comput Biol. 2014 Nov 20;10(11):e1003956. doi: 10.1371/journal.pcbi.1003956 (PMC4238950; doi:10.1371/journal.pcbi.1003956)
Supplement: Table S9 — Model variables explained by APOE e4 genotype, gender and educational level (ANOVA results). (DOCX) [file pcbi.1003956.s015.docx]

**Table S9**.

| **Demographic properties** | **Aß Production rate** ($\beta$) | **Aß Clearance rate** ($\delta$) | **Noise**  (σ) | **Onset Age**  (Age_onset_) |
| --- | --- | --- | --- | --- |
| Gender | 1.21(1.90x10^-3^) | 2.20(1.35x10^-5^) | 0.00(0.919) | 4.08(2.06x10^-9^) |
| APOE e4 | 5.51(5.38x10^-10^) | 10.48(2.24x10^-19^) | 0.40(0.224) | 13.21(1.12x10^-24^) |
| Educational Level | 0.20(0.204) | 0.32(9x10^-2^) | 0.13(0.314) | 0.00(0.876) |
| APOE e4 and Gender | 0.93(0.024) | 0.58(8x10^-2^) | 0.11(0.664) | 2.06(1.02x10^-4^) |
| APOE e4 and Educational Level | 0.13(0.302) | 0.005(0.824) | 0.74(0.019) | 0.04(0.510) |
| Gender and Educational Level | 0.06(0.771) | 0.04(0.834) | 0.09(0.697) | 0.04(0.836) |

Data are explained variance (statistical significance, i.e., P values).
