# Supplementary material for: Epidemic Spreading Model to Characterize Misfolded Proteins Propagation in Aging and Associated Neurodegenerative Disorders
Source: PLoS Comput Biol. 2014 Nov 20;10(11):e1003956. doi: 10.1371/journal.pcbi.1003956 (PMC4238950; doi:10.1371/journal.pcbi.1003956)
Supplement: Table S10 — Model variables differences between APOE e4 genotype groups (t-test results, after adjusting for gender and educational level). (DOCX) [file pcbi.1003956.s016.docx]

**Table S10**.

| **Compared groups** | **Aß Production rate** ($\beta$) | **Aß Clearance rate** ($\delta$) | **Noise**  (σ) | **Onset Age**  (Age_onset_) |
| --- | --- | --- | --- | --- |
| Non APOE e4 – APOE e4 (1 copy) | 5.78(5.47x10^-9^) | 7.46(1.35x10^-13^) | 1.57(0.06) | 8.62(0) |
| APOE e4 (1 copy) - APOE e4 (2 copies) | 0.79(0.21) | 2.73(0.003) | 0.21 (0.41) | 1.60 (0.05) |

Data are test statistic (statistical significance, i.e., P values).
