# Supplementary material for: Epidemic Spreading Model to Characterize Misfolded Proteins Propagation in Aging and Associated Neurodegenerative Disorders
Source: PLoS Comput Biol. 2014 Nov 20;10(11):e1003956. doi: 10.1371/journal.pcbi.1003956 (PMC4238950; doi:10.1371/journal.pcbi.1003956)
Supplement: Table S11 — Model variables differences between genders (t-test results, after adjusting for APOE e4 genotype and educational level). (DOCX) [file pcbi.1003956.s017.docx]

**Table S11**.

| **Compared groups** | **Aß Production rate**  ($\beta$) | **Aß Cleaning rate**  ($\delta$) | **Noise**  (σ) | **Onset Age**  (Age_onset_) |
| --- | --- | --- | --- | --- |
| Female-Male | -2.24(0.01) | -1.65(0.04) | -0.50(0.30) | -2.70(0.003) |

Data are test statistic (statistical significance, i.e., P values).
