# Supplementary material for: Epidemic Spreading Model to Characterize Misfolded Proteins Propagation in Aging and Associated Neurodegenerative Disorders
Source: PLoS Comput Biol. 2014 Nov 20;10(11):e1003956. doi: 10.1371/journal.pcbi.1003956 (PMC4238950; doi:10.1371/journal.pcbi.1003956)
Supplement: Table S12 — CSF measures explained by model variables, gender, age and educational level (ANOVA results). (DOCX) [file pcbi.1003956.s018.docx]

**Table S12**.

| **Model Variables and demographic**  **Properties** | **Aß^1-42^** | **t-tau** | **p-tau** |
| --- | --- | --- | --- |
| Aß Production rate ($\beta$) | 10.40(1.23x10^-12^) | 4.44(1.68x10^-5^) | 3.17(1.68x10^-4^) |
| Aß Clearance rate ($\delta$) | 11.84(4.83x10^-14^) | 2.77(6.31x10^-4^) | 2.37(2.00x10^-3^) |
| Noise (σ) | 0.058(0.57) | 0.39 (0.192) | 0.83(0.06) |
| Onset Age (Age_onset_) | 2.31(5.36x10^-4^) | 5.08 (4.42x10^-06^) | 4.43(2.62x10^-5^) |
| Gender | 0.80(0.04) | 0.09 (0.516) | 0.68(0.09) |
| Age | 2.96(9.30x10^-5^) | 5.44 (2.07x10^-6^) | 2.74(8.73x10^-4^) |
| Educational Level | 0.46(0.11) | 0.55 (0.122) | 1.22(0.025) |

Data are explained variance (statistical significance, i.e., P values).
